# Cancer-associated fibroblast-derived WNT5A promotes cell proliferation, metastasis, stemness and glycolysis in gastric cancer via regulating HK2

**DOI:** 10.1186/s12957-024-03482-7

**Published:** 2024-07-25

**Authors:** Yongsu Xu, Zhengju Ren, Fang Zeng, Huan Yang, Chengju Hu

**Affiliations:** 1 Nursing Department, Affiliated Hospital of Zunyi Medical University, Zunyi, China; 2 School of Nursing, Affiliated Hospital of Zunyi Medical University, Zunyi, China; 3 Hemodialysis Room, Affiliated Hospital of Zunyi Medical University, Zunyi, China; 4 Public Welfare Services Division, The Affiliated Dazu’s Hospital of Chongqing Medical University, No. 1073, South Second Ring Road, Hongxing Community, Tangxiang Street, Dazu District, Chongqing, 402360 China; 5 Health Management Center, The Affiliated Dazu’s Hospital of Chongqing Medical University, No. 1073, South Second Ring Road, Hongxing Community, Tangxiang Street, Dazu District, Chongqing, 402360 China

**Keywords:** Cancer-associated fibroblasts, Gastric cancer, WNT5A, HK2

## Abstract

**Background:**

Gastric cancer (GC) is one of the most common cancers worldwide. Tumor microenvironment plays an important role in tumor progression. This study aims to explore the role of cancer-associated fibroblasts (CAFs) in GC and the underlying mechanism.

**Methods:**

Cell viability, proliferation, invasion and migration were assessed by MTT, EdU, transwell and wound healing assays, respectively. Sphere formation assay was used to evaluate cell stemness. Glucose consumption, lactate production and ATP consumption were measured to assess glycolysis. In addition, The RNA and protein expression were detected by qRT-PCR and western blot. The interaction between wingless Type MMTV Integration Site Family, Member 5 A (WNT5A) and hexokinase 2 (HK2) was verified by Co-immunoprecipitation. The xenograft model was established to explore the function of CAFs on GC tumor growth in vivo.

**Results:**

CAFs promoted the proliferation, metastasis, stemness and glycolysis of GC cells. WNT5A was upregulated in CAFs, and CAFs enhanced WNT5A expression in GC cells. Knockdown of WNT5A in either GC cells or CAFs repressed the progression of GC cells. In addition, WNT5A promoted HK2 expression, and overexpression of HK2 reversed the effect of WNT5A knockdown in CAFs on GC cells. Besides, knockdown of WNT5A in CAFs inhibits tumor growth in vivo.

**Conclusion:**

CAF-derived WNT5A facilitates the progression of GC via regulating HK2 expression.

**Supplementary Information:**

The online version contains supplementary material available at 10.1186/s12957-024-03482-7.

## Introduction

Gastric cancer (GC), one of the most diagnosed malignancies, has long been a serious threat to public health. In recent years, the incidence of GC has decreased, but the incidence of GC is still high in elder population with the development of aging population [1]. Moreover, most GC patients are diagnosed at an advanced stage due to no obvious symptoms in the early stage, which result in a poor prognosis and a worse overall survival [2]. It will be benefited to explore the mechanism of GC tumorigenesis and progression for the improvement of diagnosis and treatment strategies for GC.

Tumor microenvironment (TME) is closely related to tumor development, and it can affect cancer cell activities, such as proliferation, migration and metabolism [3]. Cancer-associated fibroblasts (CAFs) are a vital element of TME. CAFs produce extracellular matrix (ECM) proteins and secrete growth factors, thereby contributing to cancer cell proliferation, angiogenesis, therapy resistance, and immune escape [4]. Hence, targeting CAFs has the potential to become a new cancer treatment strategy. The interaction of CAFs and cancer cells has been expounded in previous reports [4–6], and this interaction is accomplished through a variety of signaling pathways [7]. For example, CAFs regulate ROS and TGF-β signaling pathways to affect metabolism in lung cancer [8]. CAFs facilitate pancreatic cancer development and gemcitabine resistance by regulating SDF-1 mediated SATB-1 expression [9]. Besides, CAF-derived IGFBP7 enhances M2 macrophage polarization in GC via the FGF2/FGFR1/PI3K/AKT pathway [10]. However, it remains largely unclear about the mechanism of CAFs in GC progression.

It has been widely known that the Wnt signaling exerts an essential function in homeostasis and disease development, especially in cancers [11]. Wingless Type MMTV Integration Site Family, Member 5 A (WNT5A) is a ligand of the Wnt pathway and involved in various cell activities, including proliferation, migration, invasion, stemness, differentiation and polarity [12]. It has been verified that WNT5A is upregulated in multiple cancers and associated with poor prognosis. For instance, upregulated WNT5A accelerated cell metastasis in esophageal squamous cell carcinoma [13]. In addition, WNT5A also has impact on TME. A previous study revealed that WNT5A elevated GC progression through promoting IL-10 secretion and macrophage M2 polarization [14]. Study has clarified that WNT5A is highly expressed in CAFs in colorectal cancer and promotes the progression of colorectal cancer [15]. In GC, the highly expression of WNT5A in CAFs is also proved [16], while the role and mechanism of CAF-derived WNT5A remain to be explored.

Here, we verified the expression of WNT5A in GC and CAFs, and aimed to investigate the function and underlying mechanism of WNT5A as well as CAF-derived WNT5A in GC progression, which might provide new sight for the therapy for GC.

## Methods

### Patients and specimens

This project was authorized by the Ethics Committee of Affiliated Hospital of Zunyi Medical University. Twenty-nine pairs of tumor tissues and the adjacent normal tissues were obtained from GC patients during the surgery and then stored at -80℃. All GC patients enrolled in this study did not receive other treatments before surgery and signed the informed consents.

### Cell culture

Normal stomach mucosa epithelium cell line GES-1 and human GC cell lines MKN74, NCI-N87, AGS and HGC-27 cells were purchased from the Cell Bank of the Chinese Academy of Sciences (Shanghai, China) and cultured in F12K medium (only for AGS cells) or RPMI 1640 medium (Invitrogen, Carlsbad, CA, USA) with 10% fetal bovine serum (FBS, Invitrogen) and 1% penicillin/streptomycin (Gibco, Rockville, MD, USA) at 37℃ with 5% CO_2_.

CAFs from GC tissues or normal fibroblasts (NFs) from adjacent normal tissues were isolated as previously described [17]. Fibroblasts were cultured in DMEM with 10% FBS. For co-culture, fibroblasts (3–5 passages) were cultured for 48 h, and the cell medium excluding cells of was collected as conditional medium (CM). The GC cells were cultured with NF-CM or CAF-CM for 48 h for subsequent experiments.

### 3-(4,5-dimethyl-2-thiazolyl)-2,5-diphenyl-2-H-tetrazolium bromide (MTT) assay

Cell viability was detected by a MTT cell proliferation Kit (Biorigin, Beijing, China). In brief, cells in 96-well plates were added with 10 µL/well of MTT solution (5 mg/mL). Four hours later, 150 µL Formazan solubilizing buffer was added into each well. After dissolution of the crystal by oscillation, a microplate reader (Thermo Fisher Scientific, Waltham, MA, USA) was used for the absorbance at 490 nm.

### 5-ethynyl-2’-deoxyuridine (EdU) assay

Cell proliferation was assessed via an EdU kit (Beyotime, Shanghai, China). Cells were seeded into 6-well plates and incubated with EdU for 2 h. Then 4% paraformaldehyde and DAPI were used for fixation and dyeing in sequence. Finally, EDU-positive cells were photographed with a fluorescent microscope (Leica, Wetzlar, Germany).

### Transwell assay

Cell invasion was tested with the transwell chambers pre-coated with matrigel (Corning, New York, Madison, USA). The upper chambers were added with GC cells along with serum-free medium, and 700 µL medium containing 10% FBS was injected into the bottom chambers. Invaded cells were fixed using paraformaldehyde (Solarbio, Beijing, China) and dyed using 0.1% crystal violet (Solarbio) after cultured for 24 h. A microscope (Olympus, Tokyo, Japan) was used to observe the stained cells.

### Wound healing assay

Cell migration was measured by wound healing assay. The cells were seed into 6-well plates, and scratched with a 10 ul pipette tip to form wound when cells reached to 100% confluence. The cells were photographed at 0 h and 36 h.

### Sphere formation assay

Cell stemness was measured with sphere formation assay. Cells with serum free medium containing 2% B27 (Sigma, Louis, MO, USA), 20 ng/ml of epidermal growth factor (EGF, Sigma), and 20 ng/ml of basic fibroblast growth factor (bFGF, Sigma) were seeded into a low-attachment culturing 6-well plate (Corning, New York, NY, USA). At 14 days later, the spheres were formed and photographed under light microscope (Olympus).

### Cell glycolysis

Cell glycolysis was assessed by measuring glucose consumption, lactate production and ATP/ADP ratio using the glucose assay kit, lactate assay kit and ATP assay kit (Jiancheng Bioengineering Institute, Nanjing, China) according to their manufacturer’s protocols, respectively.

### Bioinformatics analysis

The differentially expressed genes in CAFs in GC were identified through the GEO database (GSE194261; https://www.ncbi.nlm.nih.gov/geo/query/acc.cgi?acc=GSE19426).

### Quantitative real-time PCR (qRT-PCR)

Total RNA was prepared by TRIzol reagent (Invitrogen), and then reversely transcription was conducted to generate cDNA using PrimeScript RT Reagent Kit (TaKaRa, Beijing, China). QPCR was performed using SYBR Green (TaKaRa) and the specific primers (Table 1). The expression of RNAs was calculated using the 2^−ΔΔCt^ method with β-actin as reference gene.

**Table 1:** Primers sequences used for PCR.

Name		Primers for PCR (5’-3’)
WNT5A	Forward	CCTCCATTCCTGGGCGCAT
	Reverse	GGACTTCTTCATGGCGAGGG
HK2	Forward	GTGAATCGGAGAGGTCCCAC
	Reverse	CAAGCAGATGCGAGGCAATC
β-actin	Forward	TGGATCAGCAAGCAGGAGTA
	Reverse	TCGGCCACATTGTGAACTTT

### Western blot

The protein was extracted following the instructions of the protein extraction kit (Beyotime). After quantified by a BCA Kit (Beyotime), the proteins were separated by SDS-PAGE and then transferred to PVDF membranes (Beyotime). The membranes were incubated with the primary antibodies including anti-WNT5A (1:2000; ab235966; abcam, Cambridge, MA, USA), anti-HK2 (1:1000; ab209847; Abcam) and anti-β-actin (1:5000; ab179467; Abcam) followed by the second antibody (1:5000; ab205718; Abcam). The blots were developed via enhanced chemiluminescence reagent (Beyotime) and analyzed using Image J software.

### Cell transfection

Small interference RNAs targeting WNT5A (si-WNT5A), HK2 overexpression vector and their controls si-NC and pcDNA were obtained from RiboBio (Guangzhou, China). SiRNA or plasmid was transfected into GC cells or CAFs using Lipofectamine 3000 (Invitrogen).

### Co-immunoprecipitation (Co-IP)

The binding between WNT5A and HK2 was verified by Co-IP. The cells were lysed and the lysates were collected. The lysates were incubated with agarose coupled anti-WNT5A, anti-HK2 or anti-IgG. Finally, the complex was eluted and the levels of proteins were detected by western blot.

### Xenograft model

The animal experiment obeyed the requirements of the Animal Care and Use Committee of Affiliated Hospital of Zunyi Medical University. Short hairpin RNA of WNT5A (sh-WNT5A) or the control (sh-NC) was transfected into CAFs. AGS cells or AGS along with CAFs containing sh-WNT5A or sh-NC were severally injected into mice (each group, *n* = 6). After 7 days, the tumor volume was detected every 3 days by measuring maximum (L) and minimum (W) lengths of the tumor and calculating using the formula: 1/2×W^2^×L. At 19 days after injection, the mice were euthanized and tumor tissues were weighed.

### Immunohistochemistry (IHC) assay

Paraffin sections were dewaxed and hydrated, and then antigen repaired by microwave, followed by serum blocking. And then the section were hatched with anti-WNT5A (1:200; ab235966; abcam), anti-HK2 (1:500; ab209847; Abcam), anti-Ki67 (1:1,000, ab15580, Abcam) or anti-MMP9 (1:1000; ab76003; Abcam), and subsequently incubated with secondary antibody. All sections were photographed under a light microscope (Olympus).

### Statistical analysis

All statistical data were analyzed with GraphPad Prism 9.0 and exhibited as mean ± standard deviation. The difference was compared using the Student’s *t*-test or analysis of variance. *P* < 0.05 meant significant difference.

## Results

### CAFs promoted the proliferation, metastasis, stemness and glycolysis of GC cells

**Fig. 1:**
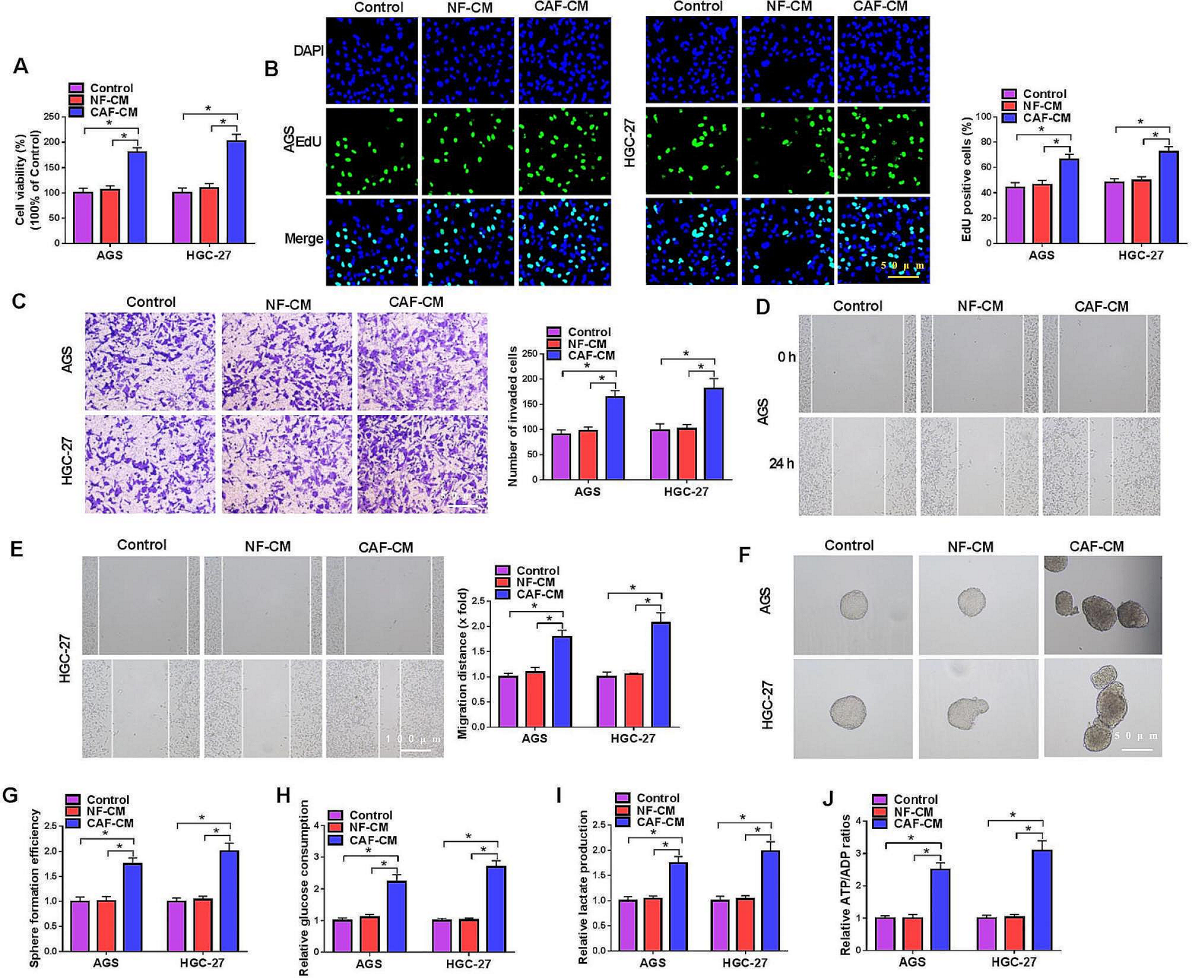
CAFs facilitated the proliferation, metastasis, stemness and glycolysis of GC cells. AGS and HGC-27 cells were incubated with the medium of NFs or CAFs, and untreated cells served as control. (**A**) The cell viability was measured by MTT assay. (**B**) Cell proliferation was assessed by EdU assay. (**C**) Cell invasion was detected by transwell assay. (**D** and **E**) Cell migration was detected by wound healing assay. (**F** and **G**) Cell stemness was assessed by sphere formation assay. (**H**-**J**) Glucose consumption, lactate production and ATP consumption were measured to assess glycolysis. *P < 0.05

Firstly, we investigated the effect of CAFs in GC cells. The result of MTT assay showed that cell viability of AGS and HGC-27 cells was enhanced after incubation with the medium of CAFs (Fig. 1A). Similarly, EdU assay proved that CAF-CM significantly facilitated GC cell proliferation (Fig. 1B). Cell invasion and migration were also promoted by CAF-CF as proved by transwell and wound healing assays (Fig. 1C-E). In addition, cell stemness was detected by sphere formation assay, which revealed that CAF-CM induced cell stemness of AGS and HGC-27 cells (Fig. 1F and G). Cell glycolysis was also tested, and the elevated levels of glucose consumption, lactate production and ATP/ADP ratio in CAF-CM group indicated that glycolysis in GC cells was expedited by CAF-CM incubation (Fig. 1H-J). Overall, these data demonstrated that CAFs contributed to the progression of GC cells.

### CAFs induced the expression of WNT5A in GC cells

**Fig. 2:**
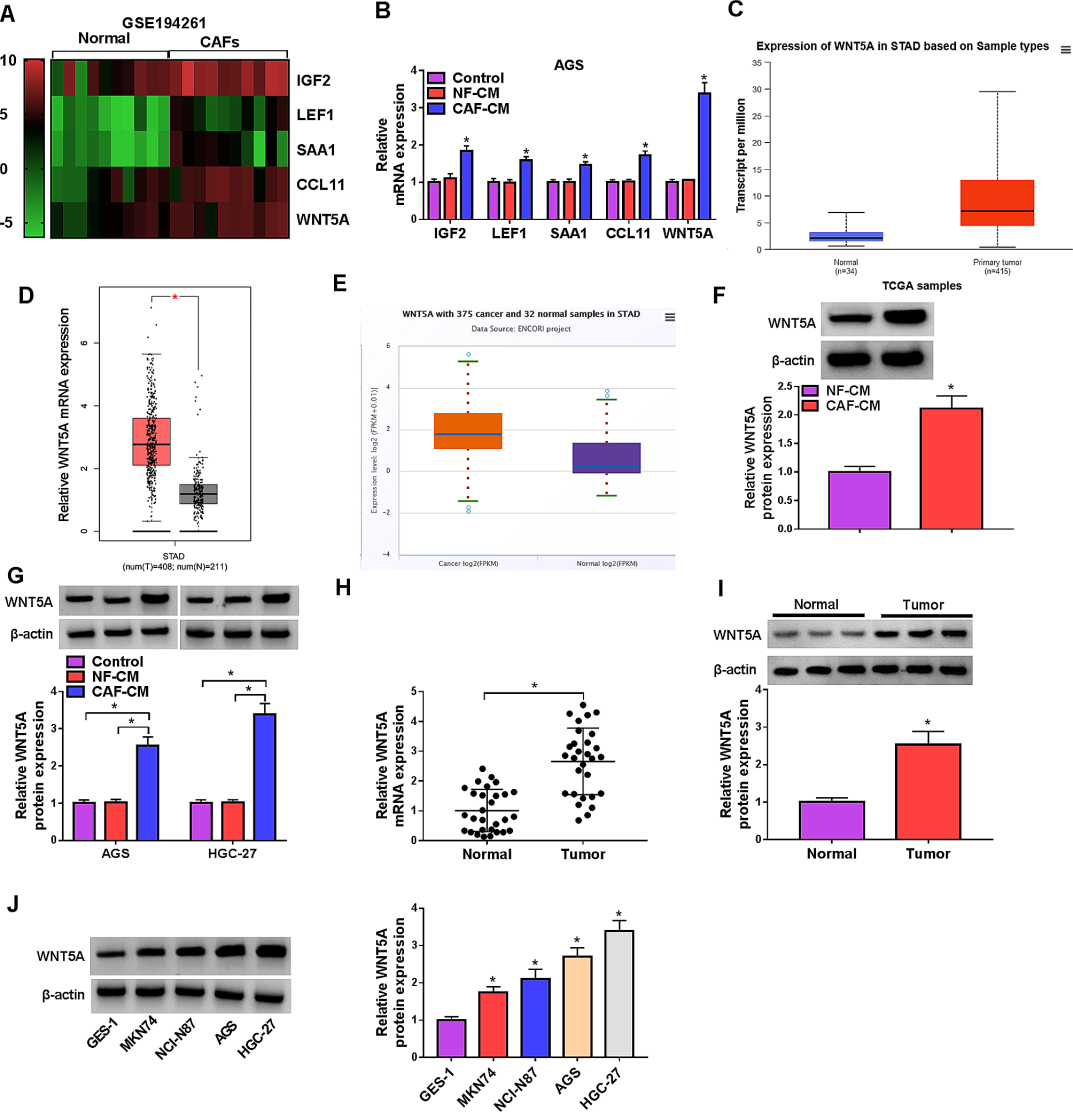
CAFs enhanced WNT5A expression in GC cells. (**A**) The expression of differentially expressed genes in CAFs in GC compared with NFs from GEO database. (**B**) The expression of differentially expressed genes was detected in AGS and HGC-27 cells incubated with the medium of NFs or CAFs by qRT-PCR. (**C**-**E**) The expression of WNT5A in GC tissues was showed from the data of TCGA, GEPIA and ENCORI databases, respectively. num(T), number of tumor tissues; num(N), number of normal tissues. (**F**) The protein expression of WNT5A was detected by western blot in the medium of NFs or CAFs. (**G**) The protein expression of WNT5A was detected by western blot in AGS and HGC-27 cells incubated with the medium of NFs or CAFs, untreated cells served as control. (**H**) The expression of WNT5A in normal and GC tissues was detected by qRT-PCR. (**I**) The protein expression of WNT5A in normal and GC tissues was detected by western blot. (**J**) The expression of WNT5A in GES-1 and GC cell was detected by western blot. *P < 0.05

According the data of the GEO database (GSE194261), a few differentially expressed genes were found in CAFs in GC compared with NFs (Fig. 2A). Subsequently, the expression of these genes was detected in AGS cells with NF-CM or CAF-CM. The data manifested that these genes were all upregulated by CAF-CM, among them, WNT5A was the most significantly upregulated gene (Fig. 2B). Therefore, WNT5A was selected for further study. The elevated expression level of WNT5A in GC tumors was also displayed in TCGA, GEPIA and ENCORI databases (Fig. 2C-E). Western blot result showed that WNT5A can be detected in both NF-CM and CAF-CM, and the WNT5A level was higher in CAF-CM than that in NF-CM (Fig. 2F). Besides, WNT5A protein expression was markedly higher in AGS and HGC-27 cells with CAF-CM than that in AGS and HGC-27 cells with control medium or NF-CM (Fig. 2G). However, CAF-CM treatment didn’t change the WNT5A level in GES-1 cells (Fig. S1). Moreover, qRT-PCR and western blot proved that WNT5A was upregulated in GC tissues and cells compared with normal tissues and GES-1 cells, respectively (Fig. 2H-J). These results manifested that WNT5A was upregulated in GC, and CAFs facilitated WNT5A expression in GC cells.

### Knockdown of WNT5A in GC cells suppressed the progression of GC cells

**Fig. 3:**
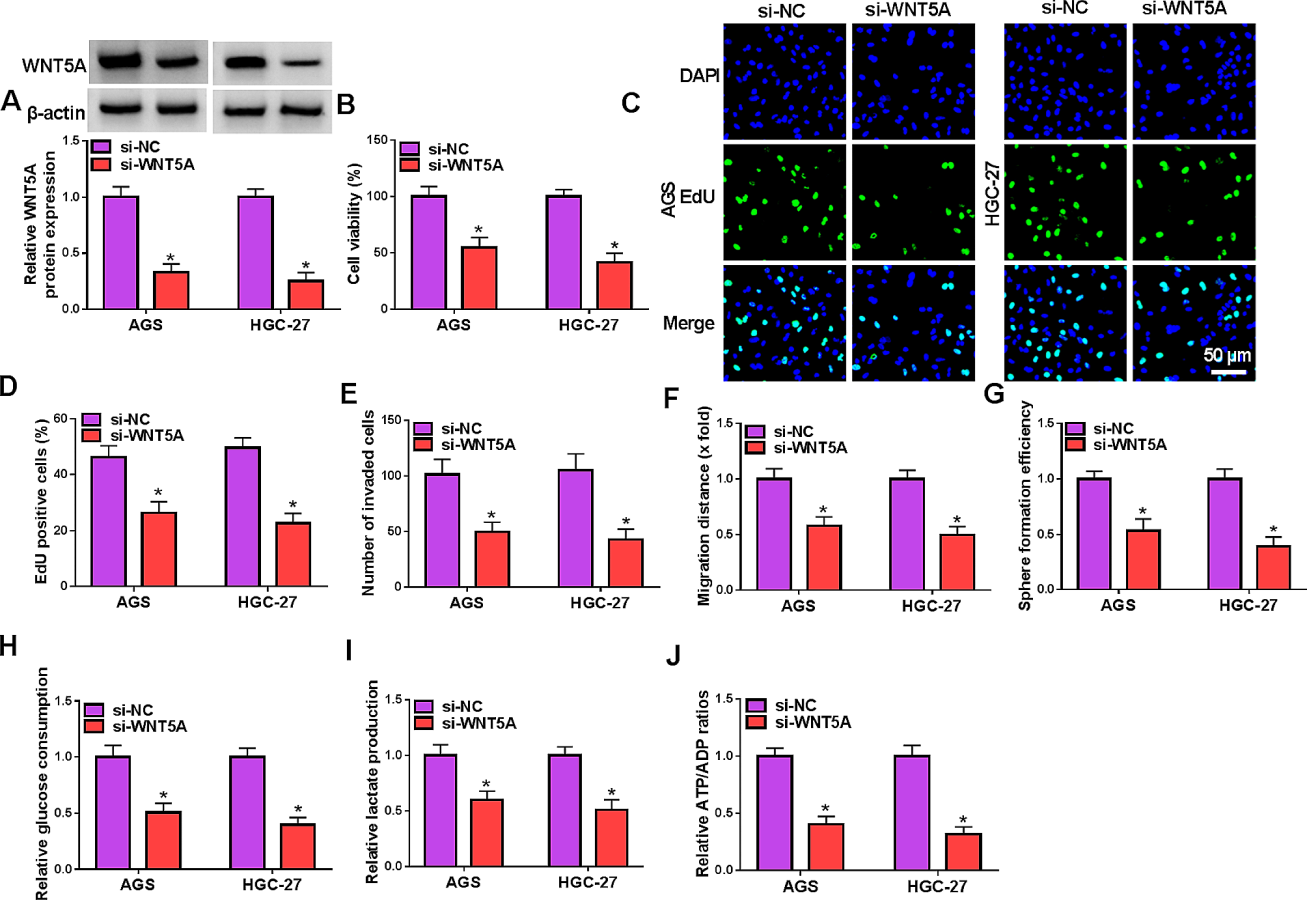
Knockdown of WNT5A in GC cells inhibited the progression of GC cells. AGS and HGC-27 cells were transfected with si-NC or si-WNT5A. (**A**) The expression of WNT5A was detected by western blot. (**B**) The cell viability was measured by MTT assay. (**C** and **D**) Cell proliferation was assessed by EdU assay. (**E**) Cell invasion was detected by transwell assay. (**F**) Cell migration was detected by wound healing assay. (**G**) Cell stemness was assessed by sphere formation assay. (**H**-**J**) Glucose consumption, lactate production and ATP consumption were measured to assess glycolysis. *P < 0.05

To explore the role of WNT5A in GC cells, WNT5A was silenced in AGS and HGC-27 cells by transfection with si-WNT5A. Western blot result showed WNT5A expressed was reduced by si-WNT5A in GC cells (Fig. 3A). MTT and EdU assays proved that the cell viability and proliferation were inhibited by WNT5A knockdown (Fig. 3B and D). As expected, knockdown of WNT5A repressed GC cell invasion and migration (Fig. 3E and F, Fig. S2A and B). Sphere formation efficiency was also decreased in GC cells transfected with si-WNT5A, suggesting that cell stemness was impeded by WNT5A knockdown (Fig. 3G, Fig. S2C). Besides, silencing WNT5A repressed GC cell glycolysis as evidenced by the decreased glucose consumption, lactate production levels and ATP/ADP ratio in si-WNT5A group (Fig. 3H-J). These results elucidated that silencing WNT5A in GC cells inhibited the progression of GC cells.

#### Knockdown of WNT5A in CAFs inhibited the progression of GC cells

**Fig. 4:**
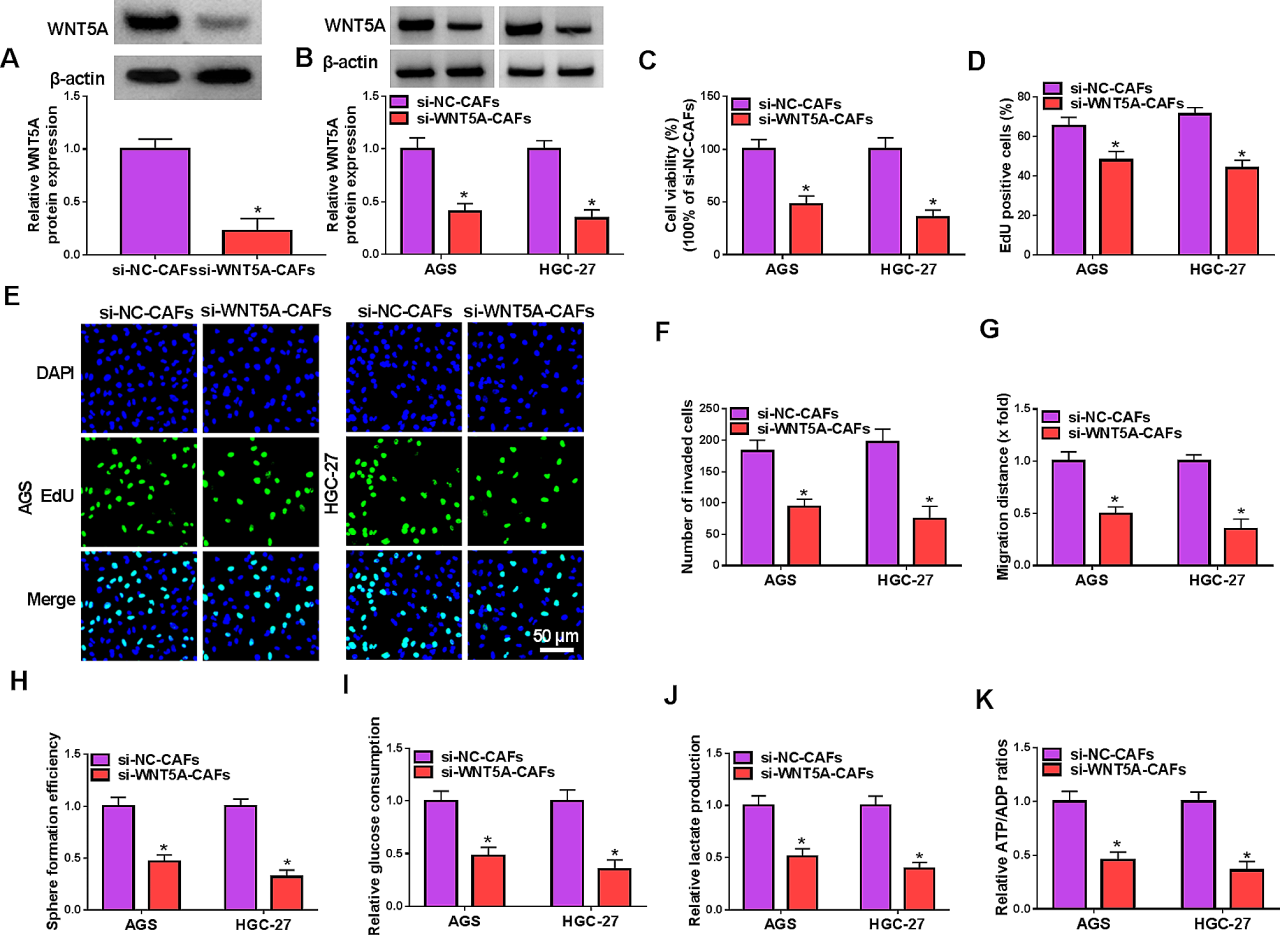
Knockdown of WNT5A in CAFs repressed the progression of GC cells. CAFs were transfected with si-NC or si-WNT5A, and then AGS and HGC-27 cells were incubated with the medium CAFs. (**A** and **B**) The expression of WNT5A was detected by western blot in CAFs with si-WNT5A and GC cells treated with si-WNT5A-CAFs. (**C**) The cell viability was measured by MTT assay. (**D** and **E**) Cell proliferation was assessed by EdU assay. (**F**) Cell invasion was detected by transwell assay. (**G**) Cell migration was detected by wound healing assay. (**H**) Cell stemness was assessed by sphere formation assay. (**I**-**K**) Glucose consumption, lactate production and ATP consumption were measured to assess glycolysis. *P < 0.05

To investigate the effect of CAF-derived WNT5A on GC cells, si-WNT5A was transfected into CAFs, and AGS and HGC-27 cells were incubated with the medium of CAFs. WNT5A protein level was reduced in CAFs with si-WNT5A and GC cells treated with si-WNT5A-CAFs (Fig. 4A and B). Knockdown of WNT5A in CAFs restrain cell viability and proliferation of GC cells (Fig. 4C-E). Meanwhile, invasion and migration of GC cells were suppressed when WNT5A was silenced in CAFs (Fig. 4F and G, Fig. S3A and B). GC cell stemness was evidently inhibited by WNT5A knockdown in CAFs (Fig. 4H, Fig. S3C). Also, WNT5A silenced CAFs retarded glycolysis of GC cells (Fig. 4I-K). These data indicated that knockdown of WNT5A in CAFs repressed GC progression.

### WNT5A positively regulated HK2 expression

**Fig. 5:**
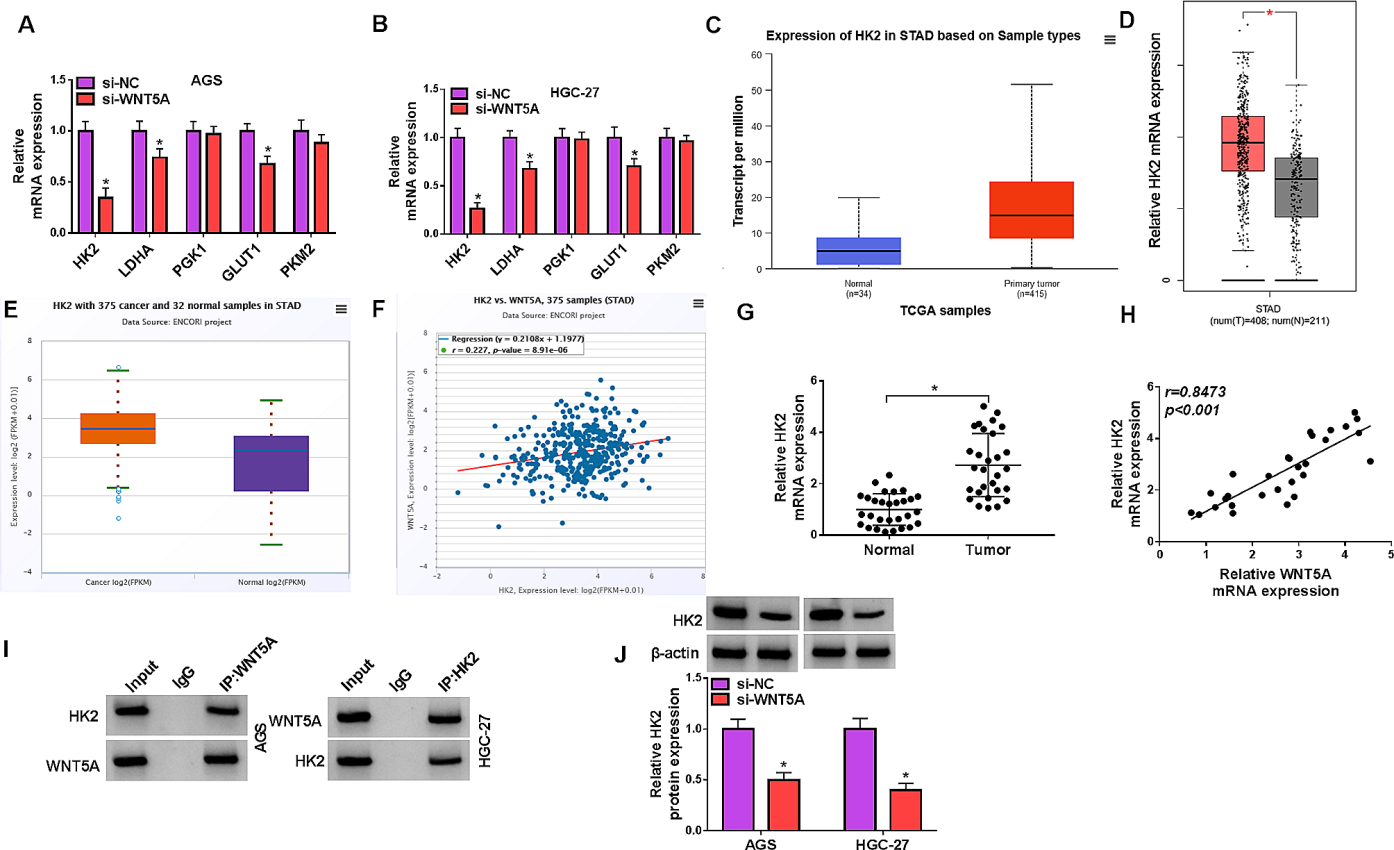
WNT5A promoted the expression HK2. (**A** and **B**) The expression of some glycolysis-related genes was detected by qRT-PCR in AGS and HGC-27 cells transfected with si-NC or si-WNT5A. (**C**-**E**) The expression of HK2 in GC tissues was showed from the data of TCGA, GEPIA and ENCORI databases, respectively. num(T), number of tumor tissues; num(N), number of normal tissues. (**F**) The correlation between WNT5A and HK2 expression in GC tissues was analyzed form the data of ENCORI databases. (**G**) The expression of HK2 in normal and GC tissues was detected by qRT-PCR. (**H**) The correlation between WNT5A and HK2 expression in GC tissues was analyzed by Pearson’s correlation coefficient assay. (**I**) The binding between WNT5A and HK2 was verified by Co-IP. (**J**) The expression of HK2 was detected by western blot in AGS and HGC-27 cells transfected with si-NC or si-WNT5A. *P < 0.05

Further, we explored the molecular mechanism of WNT5A in GC cells. AGS and HGC-27 cells were transfected with si-NC or si-WNT5A, and some glycolysis-related genes were detected. The results showed that the expression of HK2, LDHA and GLUT1 was downregulated by si-WNT5A, and HK2 was the most significantly reduced gene (Fig. 5A and B). HK2 was upregulated in GC tumor tissues according to the data in TCGA, GEPIA and ENCORI databases (Fig. 5C-E). Besides, the data of ENCORI database predicted that there was positive correlation between WNT5A and HK2 expression in GC tissues (Fig. 5F). Our qRT-PCR result showed that HK2 mRNA expression was upregulated in GC tumor tissues and positively correlated WNT5A expression (Fig. 5G and H). The data of Co-IP assay verified the binding between WNT5A and HK2 (Fig. 5I). And knockdown of WNT5A reduced HK2 protein expression in GC cells (Fig. 5J). These results implied that WNT5A interacted with HK2 and positively regulated HK2 expression in GC cells.

#### Overexpression of HK2 reversed the effects of WNT5A silenced CAFs on GC cells

**Fig. 6:**
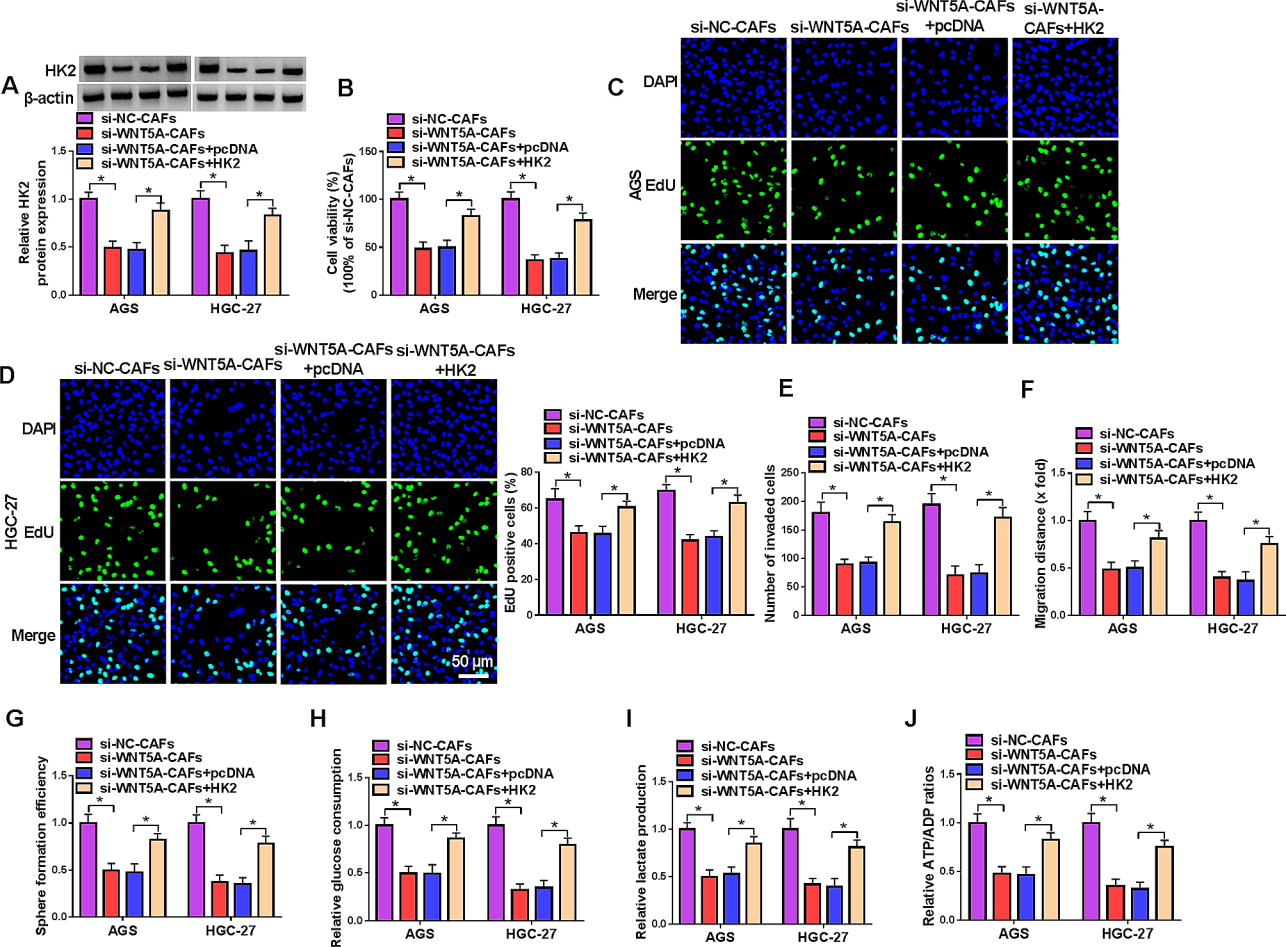
Overexpression of HK2 reversed the effects of WNT5A silenced CAFs on GC cells. CAFs were transfected with si-NC or si-WNT5A, and AGS and HGC-27 cells were transfected with pcDNA or HK2 overexpression vector and then incubated with the medium CAFs. (**A**) The expression of HK2 was detected by western blot. (**B**) The cell viability was measured by MTT assay. (**C** and **D**) Cell proliferation was assessed by EdU assay. (**E**) Cell invasion was detected by transwell assay. (**F**) Cell migration was detected by wound healing assay. (**G**) Cell stemness was assessed by sphere formation assay. (**H**-**J**) Glucose consumption, lactate production and ATP consumption were measured to assess glycolysis. *P < 0.05

Subsequently, HK2 was overexpressed in AGS and HGC-27 cells, and HK2 overexpressed GC cells were incubated with the medium of WNT5A silenced CAFs. The expression of HK2 was reduced by CAFs with WNT5A knockdown, while overexpression of HK2 reversed this effect (Fig. 6A). The inhibitory effects of WNT5A silenced CAFs on cell viability, proliferation, invasion and migration were also restored by HK2 overexpression (Fig. 6B-F, Fig. S4A and B). In addition, HK2 overexpression relieved the inhibition of WNT5A silenced CAFs on cell stemness and glycolysis (Fig. 6G-J, Fig. S4C). Besides, WNT5A overexpression CAFs enhanced cell viability, invasion and glycolysis in GC cells, and these effects were reversed by HK2 knockdown (Fig. S5). These data suggested that CAF-derived WNT5A regulated GC progression via HK2.

#### Knockdown of WNT5A in CAFs inhibited GC tumor growth

**Fig. 7:**
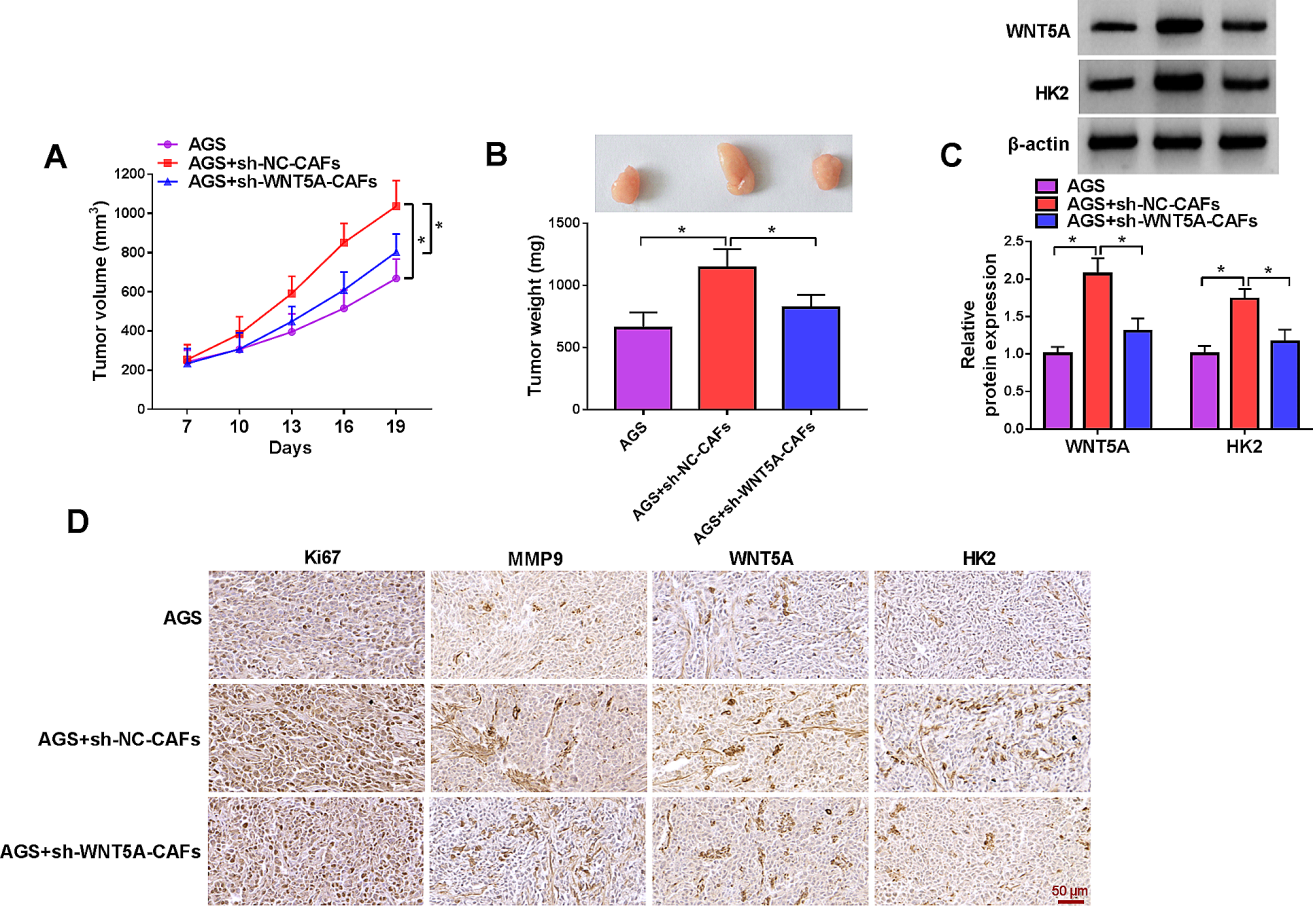
Silencing WNT5A in CAFs inhibited GC tumor growth in vivo. (**A**-**B**) Tumor volume and tumor weight were measured. (**C**) The expression of WNT5A and HK2 in mice tumors was detected by western blot. (**D**) The expression of Ki67, MMP9, WNT5A and HK2 in mice tumors was detected by IHC assay. *P < 0.05

A xenograft mouse model was established to explore the role of CAFs on GC tumor growth in vivo. Tumor volume and weight were both elevated by CAFs, but silencing WNT5A in CAFs relieved this facilitation (Fig. 7A and B). Western blot indicated that WNT5A and HK2 were upregulated by CAFs in mice tumor, while decreased when WNT5A was silenced in CAFs (Fig. 7C). Consistent with this, the IHC result showed that CAFs enhanced the expression of Ki67, MMP9, WNT5A and HK2 in vivo, which was reduced by CAFs with WNT5A knockdown (Fig. 7D). These data implicated that WNT5A mediated the facilitation of CAFs on GC tumor growth in vivo.

## Discussion

TME has a great influence on the progression, therapy and prognosis of cancer [18]. As a kind of TME cells, CAFs have attracted the attention of many researchers in recent years. Increasing studies proved that CAFs contributed to tumorigenesis and tumor progression through releasing ECM and signaling molecules [5, 19]. A previous study revealed that CAFs in GC expedited invasive activities, and were associated with poor prognosis in GC patients [20]. Besides, CAFs in GC affected immune response and promoted immune escape [21]. In our study, we verified that CAFs promoted the GC cell proliferation and metastasis, which was in accord with the previous study [22]. Cancer stem cells are self-renewal cells that accelerate cancer cell growth and metastasis, and cancer stemness reflects the malignant of the tumor to some extent [23]. It has been reported that CAFs induced stemness in bladder cancer, colorectal cancer and breast cancer [24–26]. Few studies reported the effect of CAFs on stemness in GC. Our data clarified that CAFs enhanced cell stemness in GC cells. Glycolysis is closely related with cancer development, which provides energy for cancer cells and promotes cell proliferation [27, 28]. CAFs-derived CRMP2 contributed to ovarian cancer progression via activating glycolysis pathway [29]. In non-small cell lung cancer, CAFs expedited glycolysis via activating Wnt/β-catenin signaling [30]. Similarly, the facilitation of CAFs on GC cell glycolysis was also proved in our study.

Here, we screened some differentially expressed genes in NFs and CAFs form GEO database, and found WNT5A was remarkably upregulated in CAFs in GC, which has been verified in a previous study [16]. Moreover, CAFs enhanced the expression WNT5A in GC cells. Hence, we speculated that WNT5A might mediate the function of CAFs on GC cells. WNT5A was highly expressed in GC, and high expression of WNT5A was associated with the malignant phenotype in GC [31, 32]. Knockdown of WNT5A repressed proliferation, migration and induced apoptosis of GC cells [33], and WNT5A overexpression promoted GC cell progression [34]. Consistent with these studies, we also confirmed the upregulation of WNT5A in GC cells, and loss-of-function experiments proved the tumor-promoting action of WNT5A in GC. In addition, knockdown of WNT5A in CAFs also inhibited the development of GC cells. These data confirmed our supposition.

As CAFs-derived WNT5A impeded glycolysis, we further detected some glycolysis-related genes, and found that HK2 was significantly downregulated by WNT5A. Besides, HK2 was upregulated in GC tissues and cells. As a key enzyme, HK2 catalyzes the vital rate-limiting step in glycolytic pathway [35]. CircRPS19 enhanced GC cell viability via inducing HK2-mediated glycolysis [36]. CircCUL3 upregulated transcription factor STAT3 to boost HK2 expression, thereby contributing to the progression of GC [37]. PDLIM1 interacted with HK2 and expedited GC progression via the Wnt/β-catenin signaling [38]. Our data elucidated the interaction between WNT5A and HK2, and overexpression of HK2 reverted WNT5A silenced CAFs mediated effects on GC cell progression and glycolysis. Also, CAFs-derived WNT5A elevated HK2 expression and promoted GC tumor growth in vivo. These findings suggested that CAFs-derived WNT5A regulated GC progression via HK2-mediated glycolysis.

**Fig. 8:**
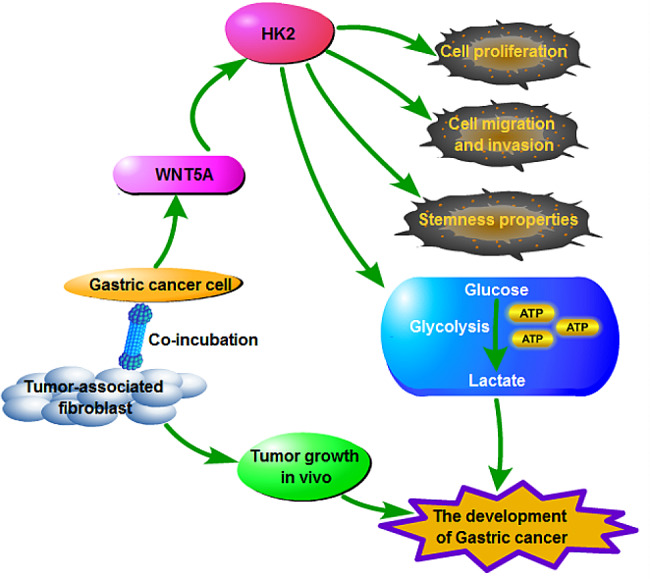
CAFs-derived WNT5A contributed to cell proliferation, metastasis, stemness and glycolysis of GC cells and promoted tumor growth in vivo via regulating HK2

In summary, this study revealed that WNT5A was highly expressed in GC, and CAFs-derived WNT5A contributed to cell proliferation, metastasis, stemness and glycolysis in GC via increasing HK2 expression (Fig. 8). Notably, our study highlighted a novel molecular mechanism of CAFs on GC progression, and indicated that CAFs might be promising therapeutic target for GC patients.

### Electronic supplementary material

Below is the link to the electronic supplementary material.

Supplementary Material 1

Supplementary Material 2

Supplementary Material 3

Supplementary Material 4

Supplementary Material 5

Supplementary Material 6

## Data Availability

The data that support the findings of this study are available from the corresponding author upon reasonable request.

## References

[1] Smyth EC, Nilsson M, Grabsch HI, van Grieken NC, Lordick F. Gastric cancer. Lancet. 2020;396(10251):635–48. 32861308 10.1016/S0140-6736(20)31288-5

[2] Lazăr DC, Avram MF, Romoșan I, Cornianu M, Tăban S, Goldiș A. Prognostic significance of tumor immune microenvironment and immunotherapy: novel insights and future perspectives in gastric cancer. World J Gastroenterol. 2018;24(32):3583–616. 30166856 10.3748/wjg.v24.i32.3583 PMC6113718

[3] Hinshaw DC, Shevde LA. The Tumor Microenvironment innately modulates Cancer Progression. Cancer Res. 2019;79(18):4557–66. 31350295 10.1158/0008-5472.CAN-18-3962 PMC6744958

[4] Sahai E, Astsaturov I, Cukierman E, DeNardo DG, Egeblad M, Evans RM, et al. A framework for advancing our understanding of cancer-associated fibroblasts. Nat Rev Cancer. 2020;20(3):174–86. 31980749 10.1038/s41568-019-0238-1 PMC7046529

[5] Chen X, Song E. Turning foes to friends: targeting cancer-associated fibroblasts. Nat Rev Drug Discov. 2019;18(2):99–115. 30470818 10.1038/s41573-018-0004-1

[6] Ishii G, Ochiai A, Neri S. Phenotypic and functional heterogeneity of cancer-associated fibroblast within the tumor microenvironment. Adv Drug Deliv Rev. 2016;99(Pt B):186–96. 26278673 10.1016/j.addr.2015.07.007

[7] Fang Z, Meng Q, Xu J, Wang W, Zhang B, Liu J, et al. Signaling pathways in cancer-associated fibroblasts: recent advances and future perspectives. Cancer Commun (Lond). 2023;43(1):3–41. 36424360 10.1002/cac2.12392 PMC9859735

[8] Cruz-Bermúdez A, Laza-Briviesca R, Vicente-Blanco RJ, García-Grande A, Coronado MJ, Laine-Menéndez S, et al. Cancer-associated fibroblasts modify lung cancer metabolism involving ROS and TGF-β signaling. Free Radic Biol Med. 2019;130:163–73. 30391585 10.1016/j.freeradbiomed.2018.10.450

[9] Wei L, Ye H, Li G, Lu Y, Zhou Q, Zheng S, et al. Cancer-associated fibroblasts promote progression and gemcitabine resistance via the SDF-1/SATB-1 pathway in pancreatic cancer. Cell Death Dis. 2018;9(11):1065. 30337520 10.1038/s41419-018-1104-x PMC6194073

[10] Li D, Xia L, Huang P, Wang Z, Guo Q, Huang C, et al. Cancer-associated fibroblast-secreted IGFBP7 promotes gastric cancer by enhancing tumor associated macrophage infiltration via FGF2/FGFR1/PI3K/AKT axis. Cell Death Discov. 2023;9(1):17. 36681667 10.1038/s41420-023-01336-x PMC9867714

[11] Asem MS, Buechler S, Wates RB, Miller DL, Stack MS. Wnt5a Signaling in Cancer. Cancers (Basel). 2016;8(9):79. 27571105 10.3390/cancers8090079 PMC5040981

[12] Bueno MLP, Saad STO, Roversi FM. WNT5A in tumor development and progression: a comprehensive review. Biomed Pharmacother. 2022;155113599. 10.1016/j.biopha.2022.113599 36089446

[13] Feng Y, Ma Z, Pan M, Xu L, Feng J, Zhang Y, et al. WNT5A promotes the metastasis of esophageal squamous cell carcinoma by activating the HDAC7/SNAIL signaling pathway. Cell Death Dis. 2022;13(5):480. 35595735 10.1038/s41419-022-04901-x PMC9122958

[14] Liu Q, Yang C, Wang S, Shi D, Wei C, Song J, et al. Wnt5a-induced M2 polarization of tumor-associated macrophages via IL-10 promotes colorectal cancer progression. Cell Commun Signal. 2020;18(1):51. 32228612 10.1186/s12964-020-00557-2 PMC7106599

[15] Hirashima T, Karasawa H, Aizawa T, Suzuki T, Yamamura A, Suzuki H, et al. Wnt5a in cancer-associated fibroblasts promotes colorectal cancer progression. Biochem Biophys Res Commun. 2021;568:37–42. 34175688 10.1016/j.bbrc.2021.06.062

[16] Rogers S, Zhang C, Anagnostidis V, Liddle C, Fishel ML, Gielen F, et al. Cancer-associated fibroblasts influence Wnt/PCP signaling in gastric cancer cells by cytoneme-based dissemination of ROR2. Proc Natl Acad Sci U S A. 2023;120(39):e2217612120. 37722040 10.1073/pnas.2217612120 PMC10523461

[17] Yasuda T, Koiwa M, Yonemura A, Akiyama T, Baba H, Ishimoto T. Protocol to establish cancer-associated fibroblasts from surgically resected tissues and generate senescent fibroblasts. STAR Protoc. 2021;2(2):100553. 34136831 10.1016/j.xpro.2021.100553 PMC8176369

[18] Bilotta MT, Antignani A, Fitzgerald DJ. Managing the TME to improve the efficacy of cancer therapy. Front Immunol. 2022;13954992. 10.3389/fimmu.2022.954992 PMC9630343 36341428

[19] Higashino N, Koma YI, Hosono M, Takase N, Okamoto M, Kodaira H, et al. Fibroblast activation protein-positive fibroblasts promote tumor progression through secretion of CCL2 and interleukin-6 in esophageal squamous cell carcinoma. Lab Invest. 2019;99(6):777–92. 30683902 10.1038/s41374-018-0185-6

[20] Li X, Sun Z, Peng G, Xiao Y, Guo J, Wu B, et al. Single-cell RNA sequencing reveals a pro-invasive cancer-associated fibroblast subgroup associated with poor clinical outcomes in patients with gastric cancer. Theranostics. 2022;12(2):620–38. 34976204 10.7150/thno.60540 PMC8692898

[21] Mak TK, Li X, Huang H, Wu K, Huang Z, He Y et al. The cancer-associated fibroblast-related signature predicts prognosis and indicates immune microenvironment infiltration in gastric cancer. Front Immunol. 2022;13951214. 10.3389/fimmu.2022.951214 PMC9372353 35967313

[22] Pang T, Yin X, Luo T, Lu Z, Nie M, Yin K, et al. Cancer-associated fibroblasts promote malignancy of gastric cancer cells via Nodal signalling. Cell Biochem Funct. 2020;38(1):4–11. 31733068 10.1002/cbf.3446

[23] Chen P, Hsu WH, Han J, Xia Y, DePinho RA. Cancer Stemness meets immunity: from mechanism to Therapy. Cell Rep. 2021;34(1):108597. 33406434 10.1016/j.celrep.2020.108597 PMC7839836

[24] Ma Z, Li X, Mao Y, Wei C, Huang Z, Li G, et al. Interferon-dependent SLC14A1(+) cancer-associated fibroblasts promote cancer stemness via WNT5A in bladder cancer. Cancer Cell. 2022;40(12):1550–e15651557. 36459995 10.1016/j.ccell.2022.11.005

[25] Hu JL, Wang W, Lan XL, Zeng ZC, Liang YS, Yan YR, et al. CAFs secreted exosomes promote metastasis and chemotherapy resistance by enhancing cell stemness and epithelial-mesenchymal transition in colorectal cancer. Mol Cancer. 2019;18(1):91. 31064356 10.1186/s12943-019-1019-x PMC6503554

[26] Ren J, Smid M, Iaria J, Salvatori DCF, van Dam H, Zhu HJ, et al. Cancer-associated fibroblast-derived Gremlin 1 promotes breast cancer progression. Breast Cancer Res. 2019;21(1):109. 31533776 10.1186/s13058-019-1194-0 PMC6751614

[27] Abbaszadeh Z, Çeşmeli S, Biray Avcı Ç. Crucial players in glycolysis: Cancer progress. Gene. 2020;726144158. 10.1016/j.gene.2019.144158 31629815

[28] Chelakkot C, Chelakkot VS, Shin Y, Song K. Modulating glycolysis to Improve Cancer Therapy. Int J Mol Sci. 2023;24(3):2606. 36768924 10.3390/ijms24032606 PMC9916680

[29] Jin Y, Bian S, Wang H, Mo J, Fei H, Li L, et al. CRMP2 derived from cancer associated fibroblasts facilitates progression of ovarian cancer via HIF-1α-glycolysis signaling pathway. Cell Death Dis. 2022;13(8):675. 35927239 10.1038/s41419-022-05129-5 PMC9352901

[30] Zhang H, Zhang K, Qiu L, Yue J, Jiang H, Deng Q, et al. Cancer-associated fibroblasts facilitate DNA damage repair by promoting the glycolysis in non-small cell lung cancer. Biochim Biophys Acta Mol Basis Dis. 2023;1869(5):166670. 36822449 10.1016/j.bbadis.2023.166670

[31] Hu Q, Li LL, Peng Z, Yi P. Expression of hepatocyte nuclear factor 4 alpha, wingless-related integration site, and β-catenin in clinical gastric cancer. World J Clin Cases. 2022;10(21):7242–55. 36157990 10.12998/wjcc.v10.i21.7242 PMC9353908

[32] Nam S, Chung JW, Yang JY. WNT5A correlates with clinicopathological characteristics in gastric Cancer: a Meta-analysis. Cell Physiol Biochem. 2017;41(1):33–40. 28135710 10.1159/000455934

[33] Xu Z, Yu Z, Tan Q, Wei C, Tang Q, Wang L, et al. MiR-876-5p regulates gastric cancer cell proliferation, apoptosis and migration through targeting WNT5A and MITF. Biosci Rep. 2019;39(6):BSR20190066. 31171711 10.1042/BSR20190066 PMC6597843

[34] Gao M, Liu L, Yang Y, Li M, Ma Q, Chang Z. LncRNA HCP5 induces gastric Cancer Cell Proliferation, Invasion, and EMT processes through the miR-186-5p/WNT5A Axis under Hypoxia. Front Cell Dev Biol. 2021;9663654. 10.3389/fcell.2021.663654 PMC8226141 34178988

[35] Ros S, Schulze A. Glycolysis back in the limelight: systemic targeting of HK2 blocks tumor growth. Cancer Discov. 2013;3(10):1105–7. 24124231 10.1158/2159-8290.CD-13-0565

[36] Zheng X, Shao J, Qian J, Liu S. circRPS19 affects HK2–mediated aerobic glycolysis and cell viability via the miR–125a–5p/USP7 pathway in gastric cancer. Int J Oncol. 2023;63(2):98. 37449524 10.3892/ijo.2023.5546 PMC10552706

[37] Pu Z, Xu M, Yuan X, Xie H, Zhao J. Circular RNA circCUL3 accelerates the Warburg Effect Progression of Gastric Cancer through regulating the STAT3/HK2 Axis. Mol Ther Nucleic Acids. 2020;22310–318. 10.1016/j.omtn.2020.08.023 PMC7527579 33230436

[38] Lei Y, He L, Li Y, Hou J, Zhang H, Li G. PDLIM1 interacts with HK2 to promote gastric cancer progression through enhancing the Warburg effect via Wnt/β-catenin signaling. Cell Tissue Res. 2023. 10.1007/s00441-023-03840-z 37930472

