# Supplementary material for: Cancer-associated fibroblast-derived WNT5A promotes cell proliferation, metastasis, stemness and glycolysis in gastric cancer via regulating HK2
Source: World J Surg Oncol. 2024 Jul 25;22:193. doi: 10.1186/s12957-024-03482-7 (PMC11270928; doi:10.1186/s12957-024-03482-7)

Figure 2F

WNT5A

β-actin

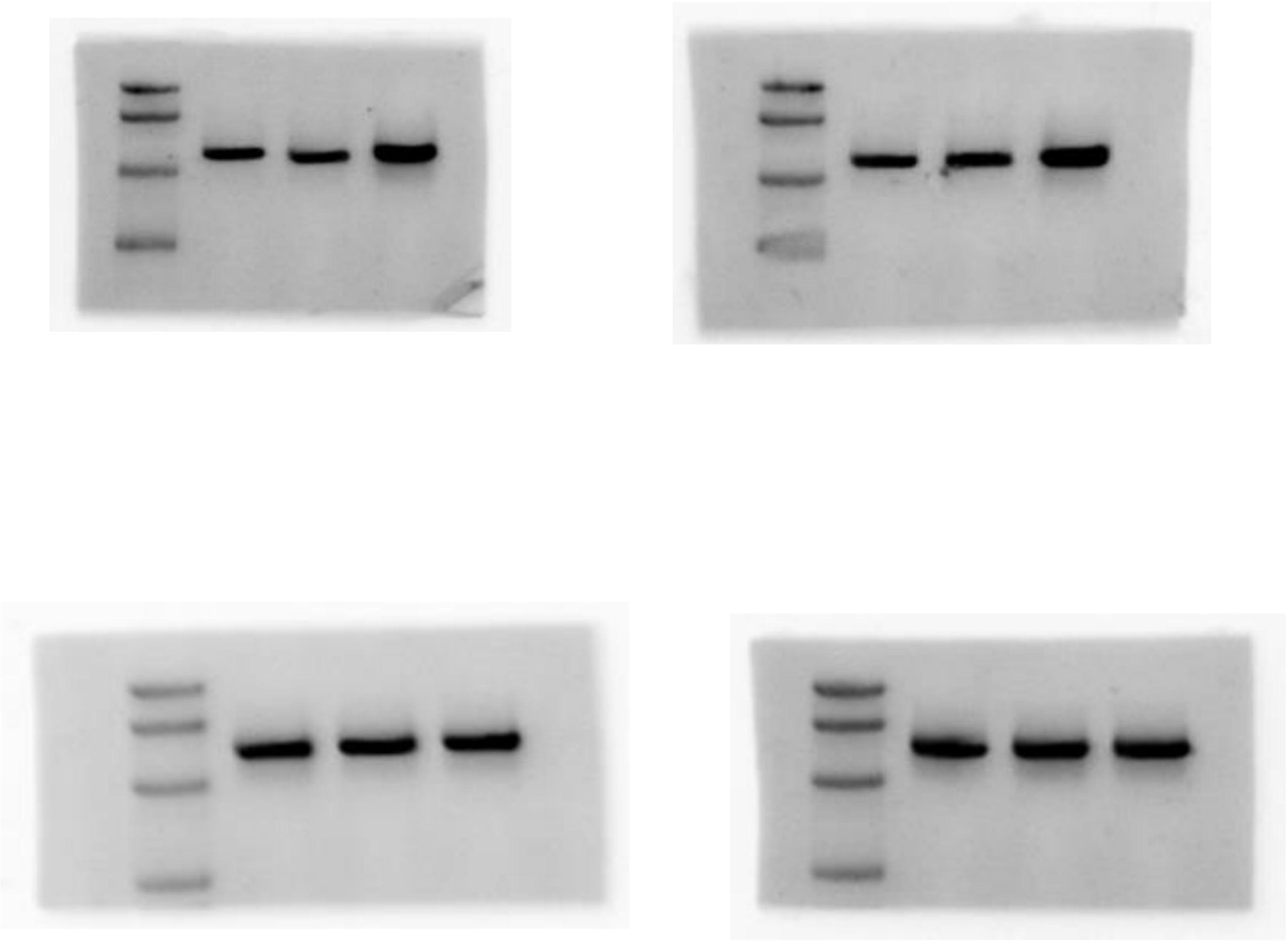

Figure 2H

WNT5A

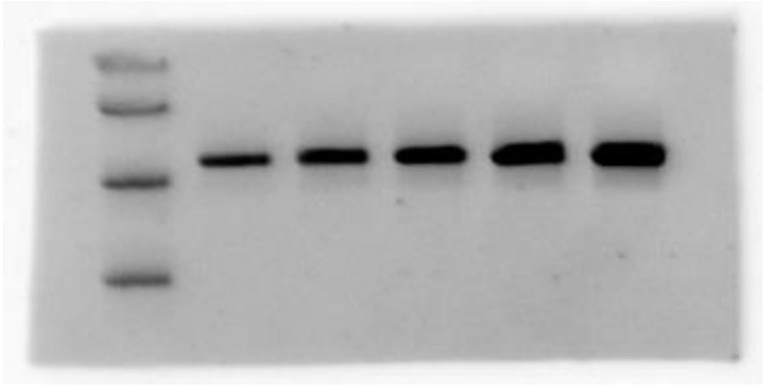

β-actin

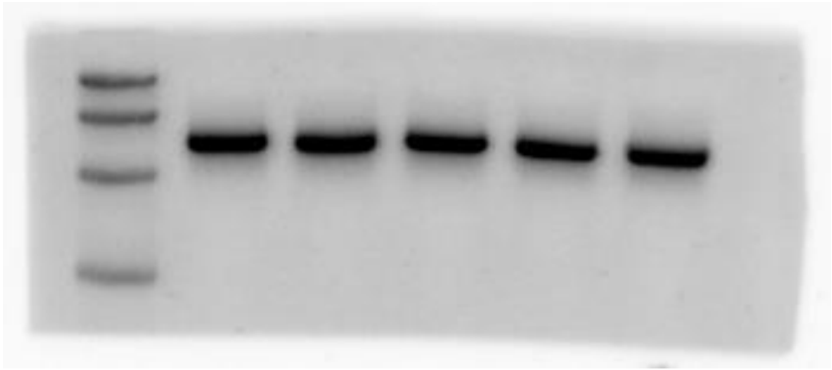

Figure 3A

WNT5A

β-actin

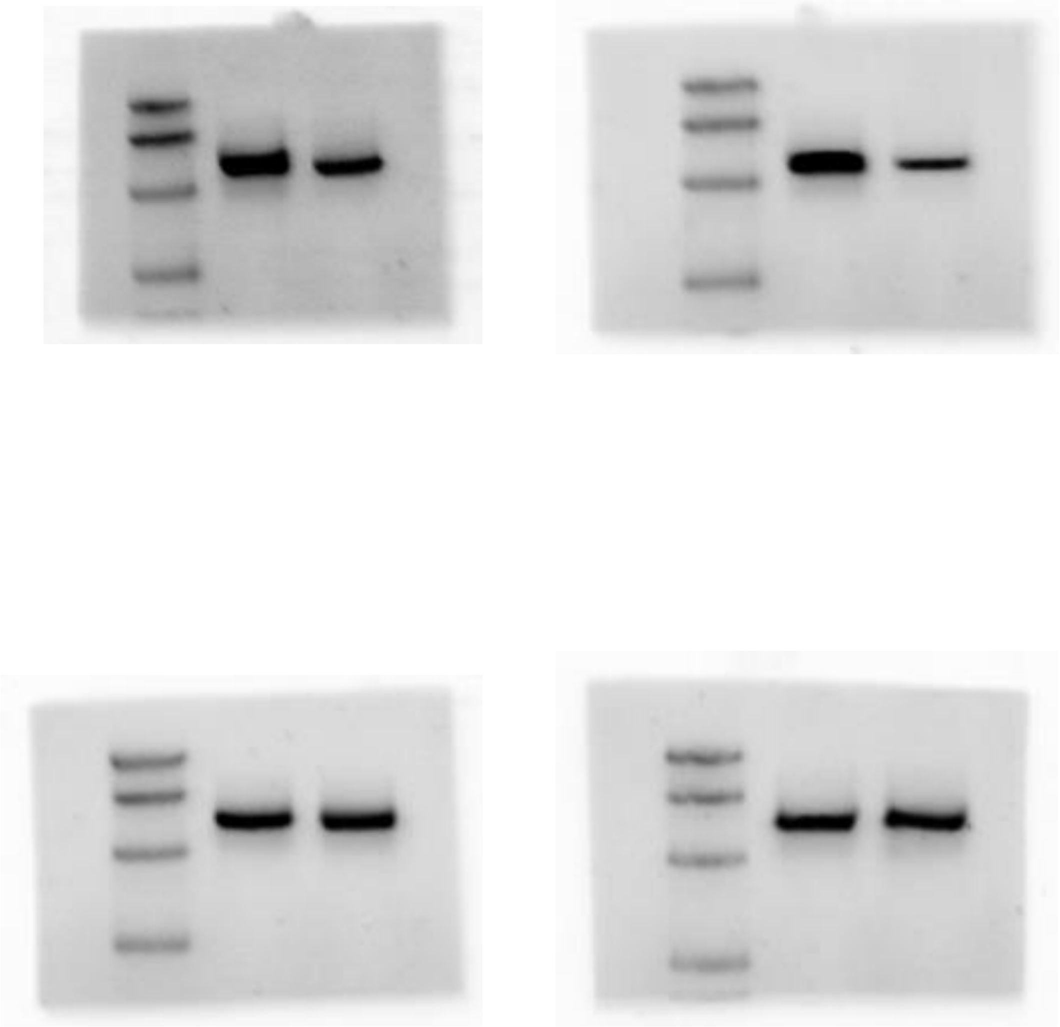

Figure 4A

WNT5A

β-actin

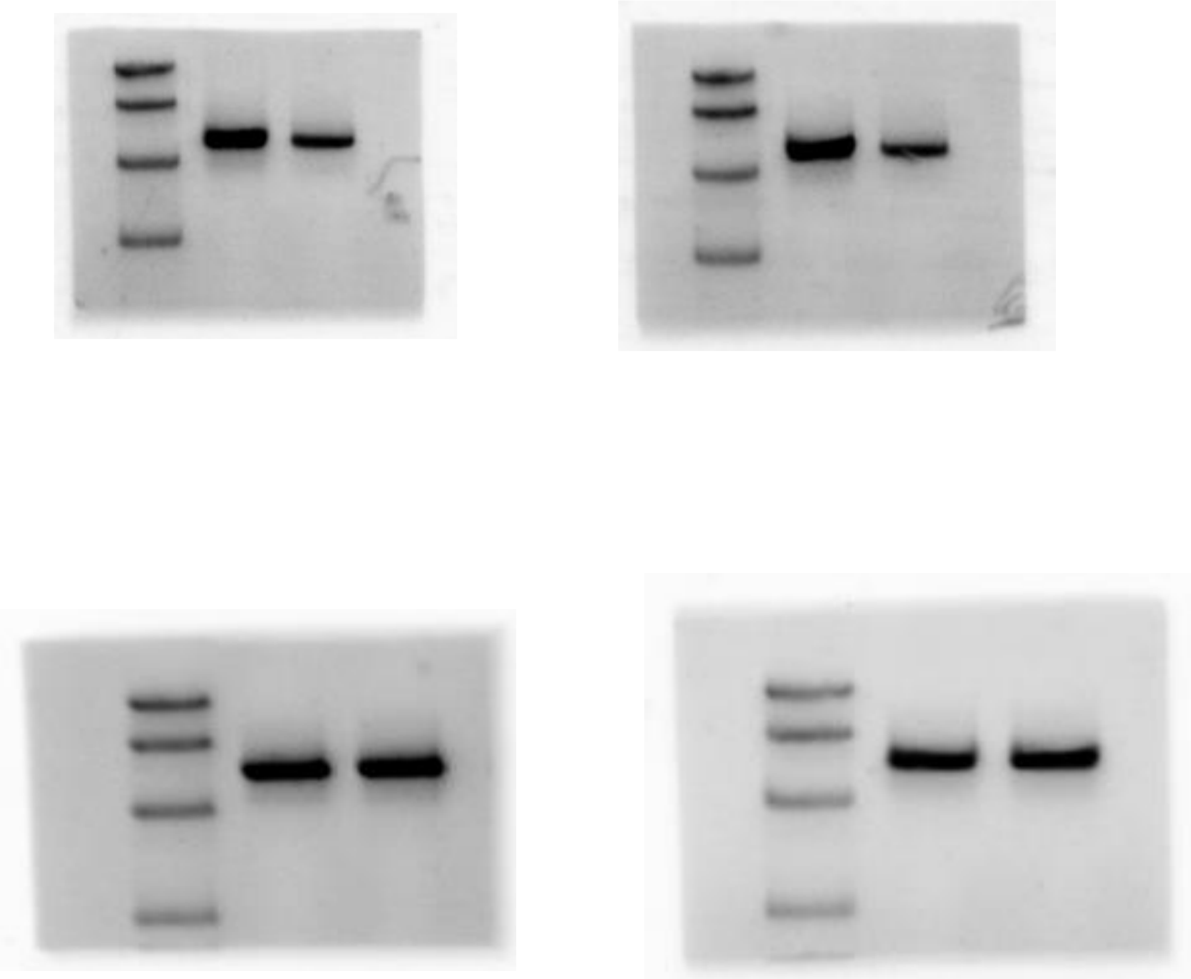

Figure 5I

HK2

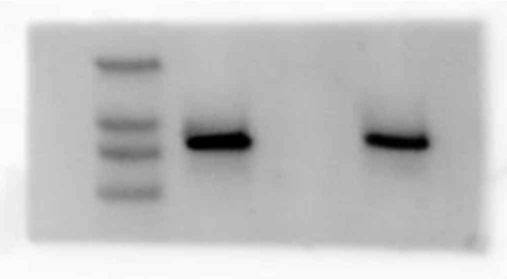

WNT5A

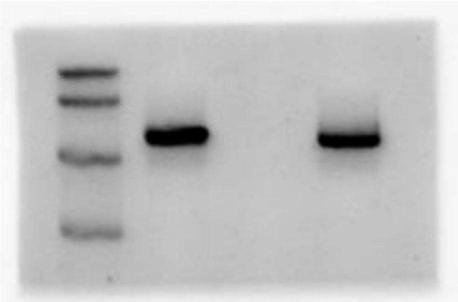

WNT5A

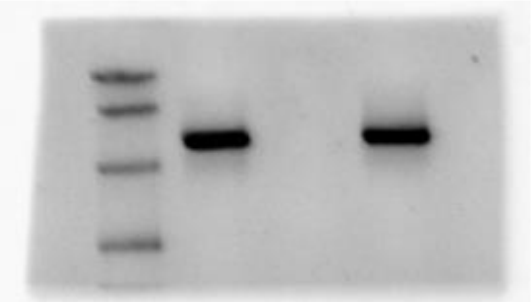

HK2

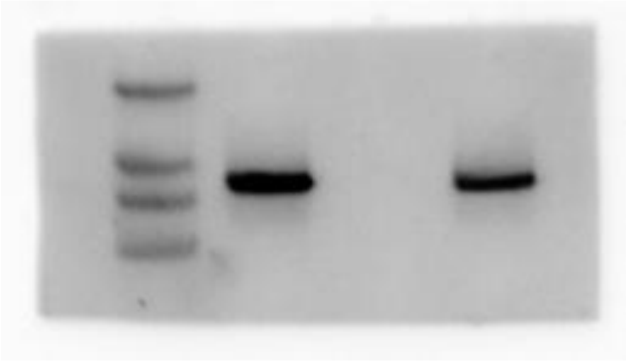

Figure 5J

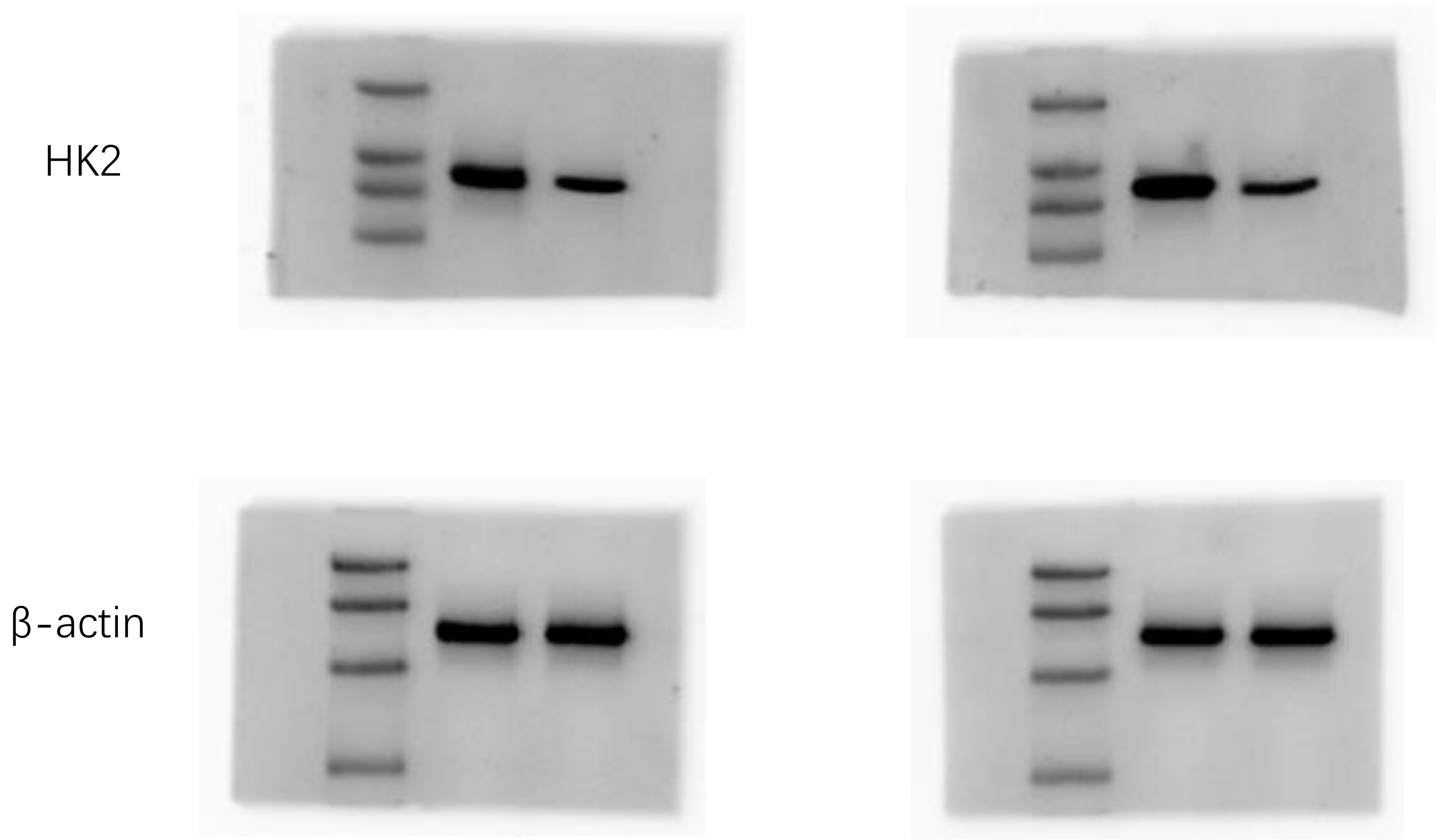

Figure 6A

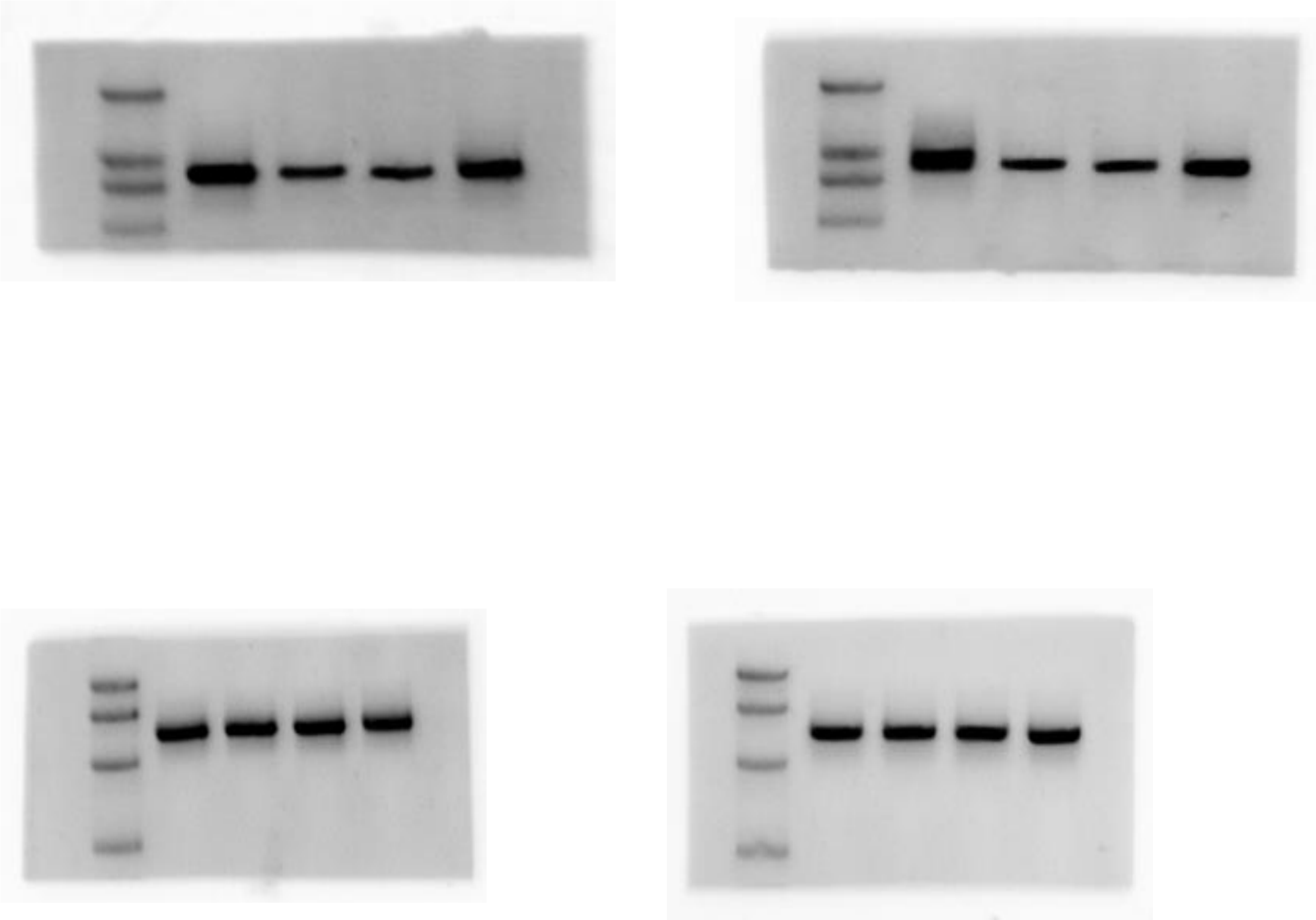

Figure 7C

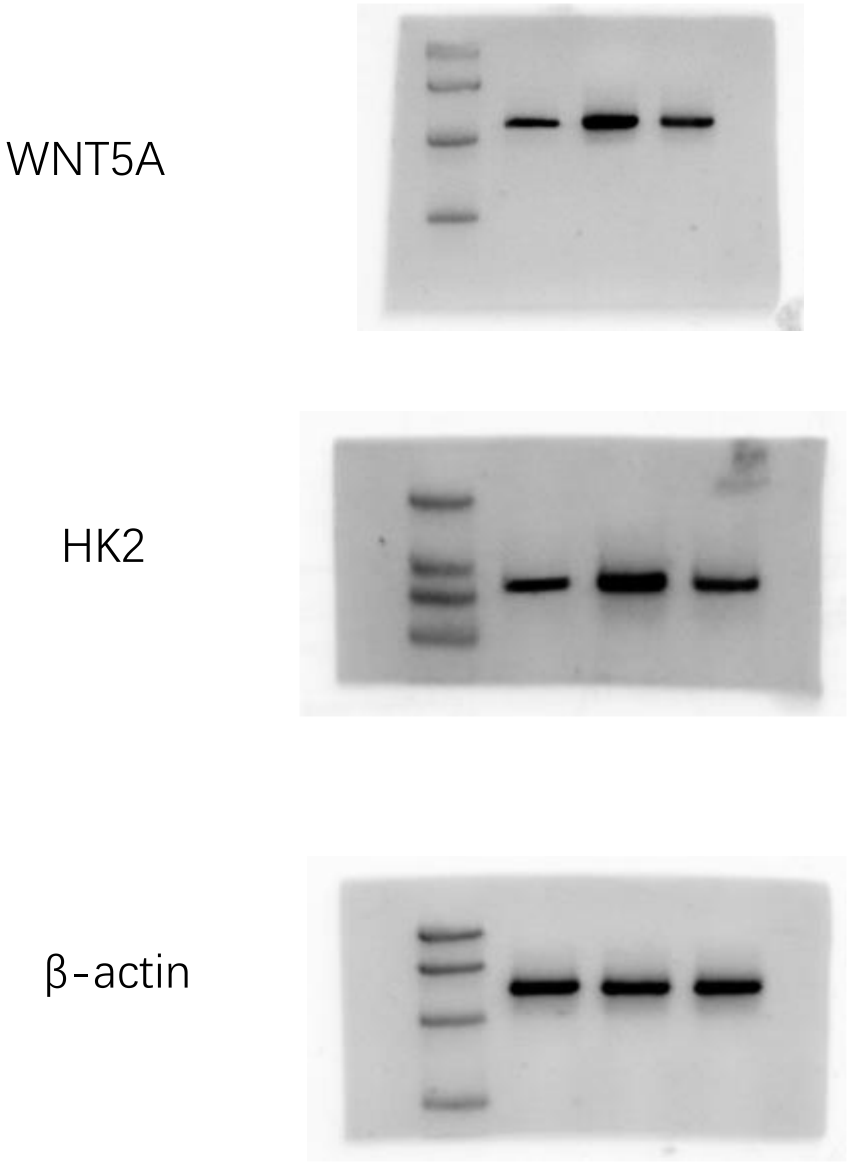

Supplement: Supplementary file 1 — Supplementary Material 1 [file 12957_2024_3482_MOESM1_ESM.pdf]
