# Supplementary figures and images for: Cancer-associated fibroblast-derived WNT5A promotes cell proliferation, metastasis, stemness and glycolysis in gastric cancer via regulating HK2
Source: World J Surg Oncol. 2024 Jul 25;22:193. doi: 10.1186/s12957-024-03482-7 (PMC11270928; doi:10.1186/s12957-024-03482-7)

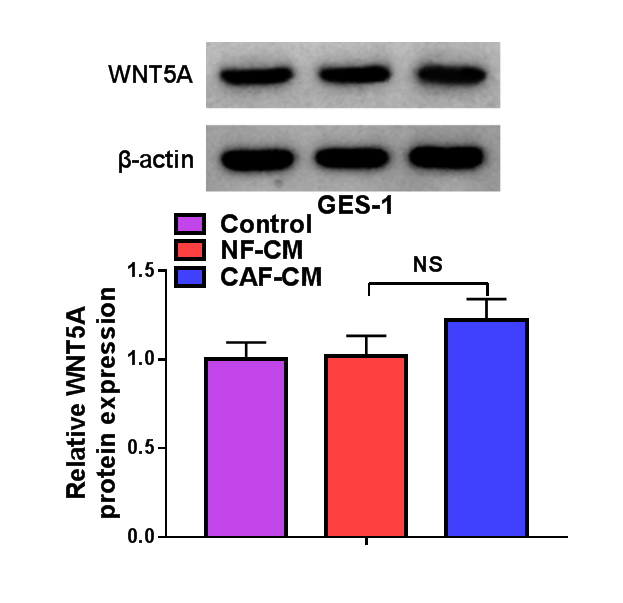

Supplement: Supplementary file 2 — Supplementary Material 2 [file 12957_2024_3482_MOESM2_ESM.png]

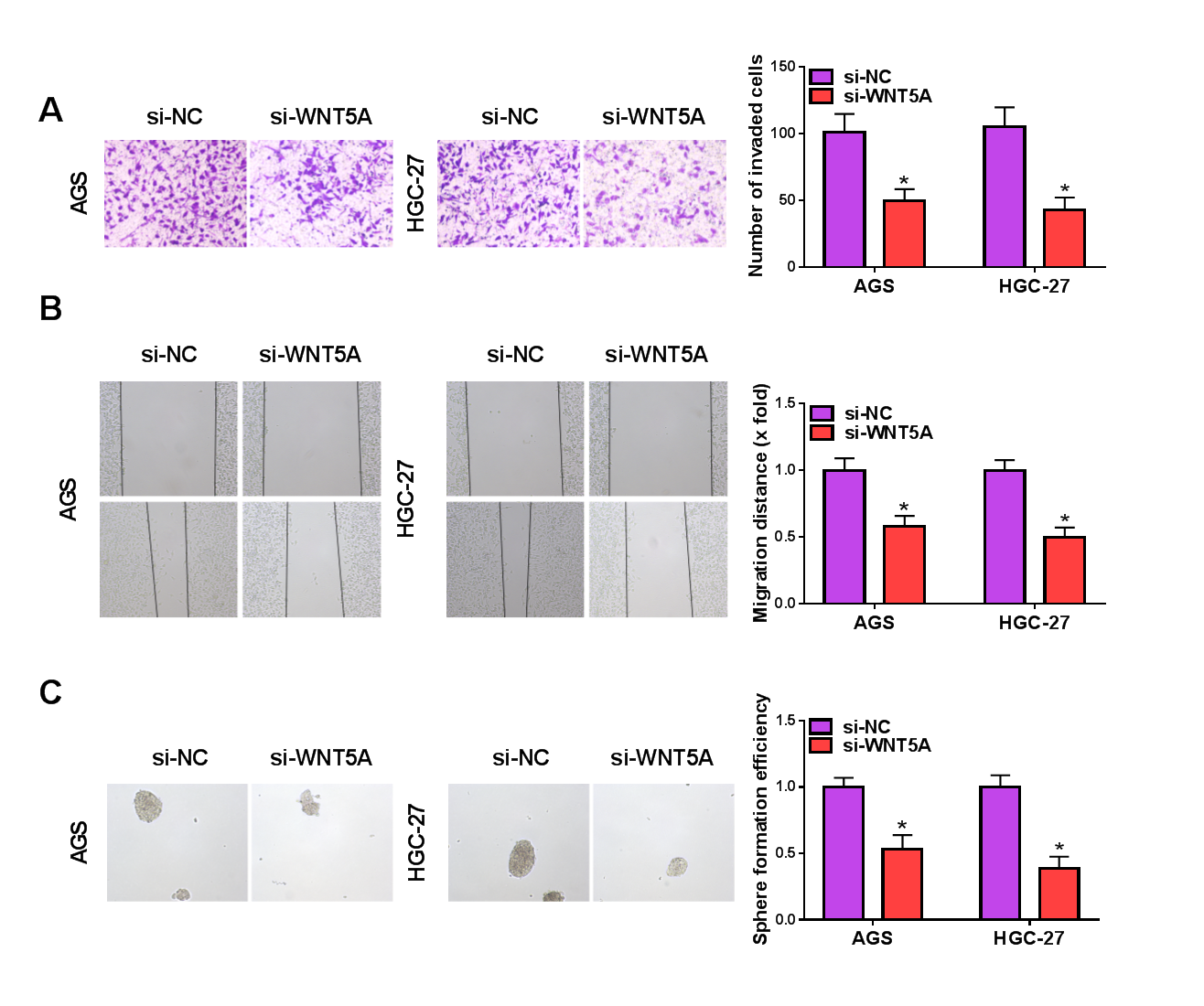

Supplement: Supplementary file 3 — Supplementary Material 3 [file 12957_2024_3482_MOESM3_ESM.png]

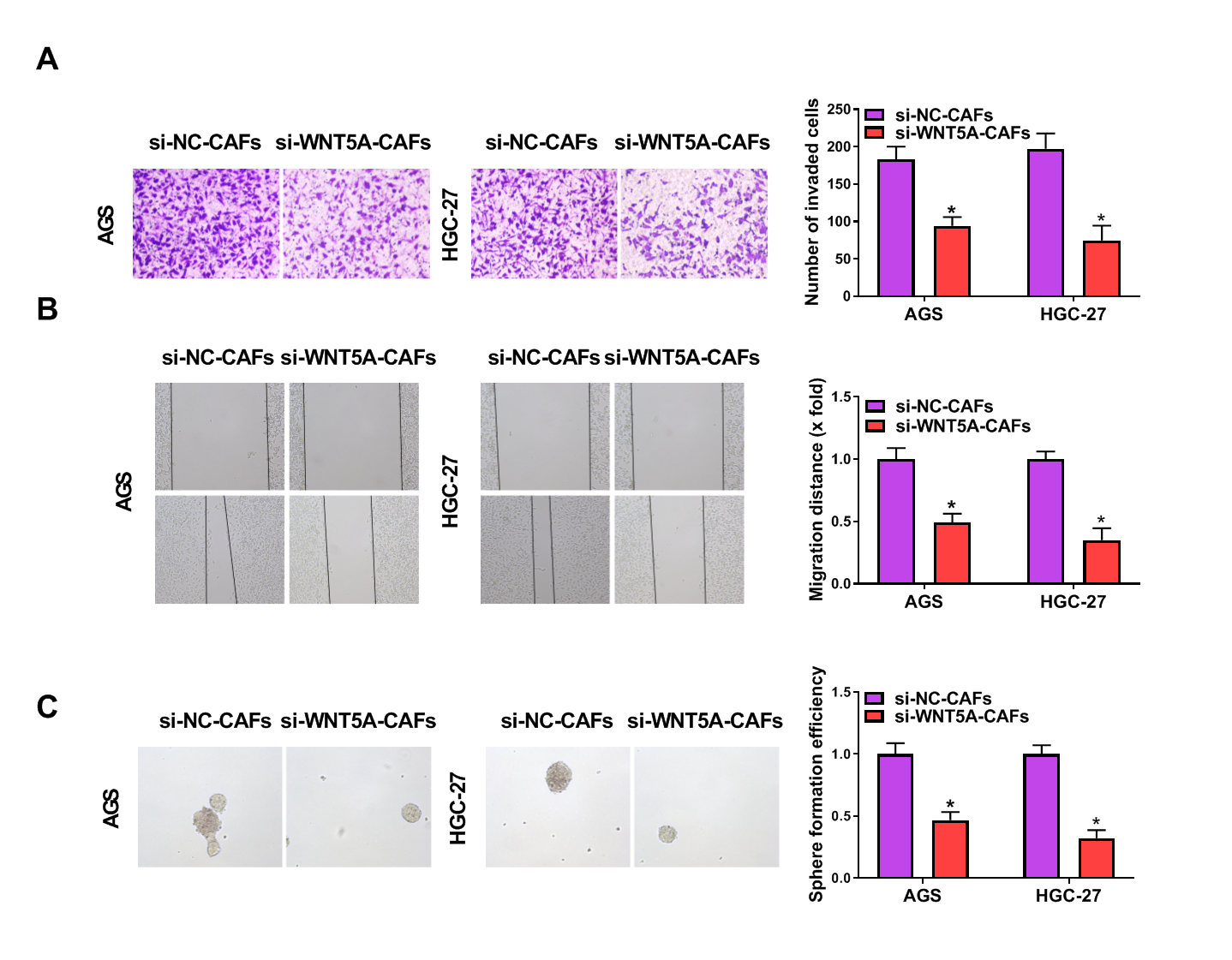

Supplement: Supplementary file 4 — Supplementary Material 4 [file 12957_2024_3482_MOESM4_ESM.png]

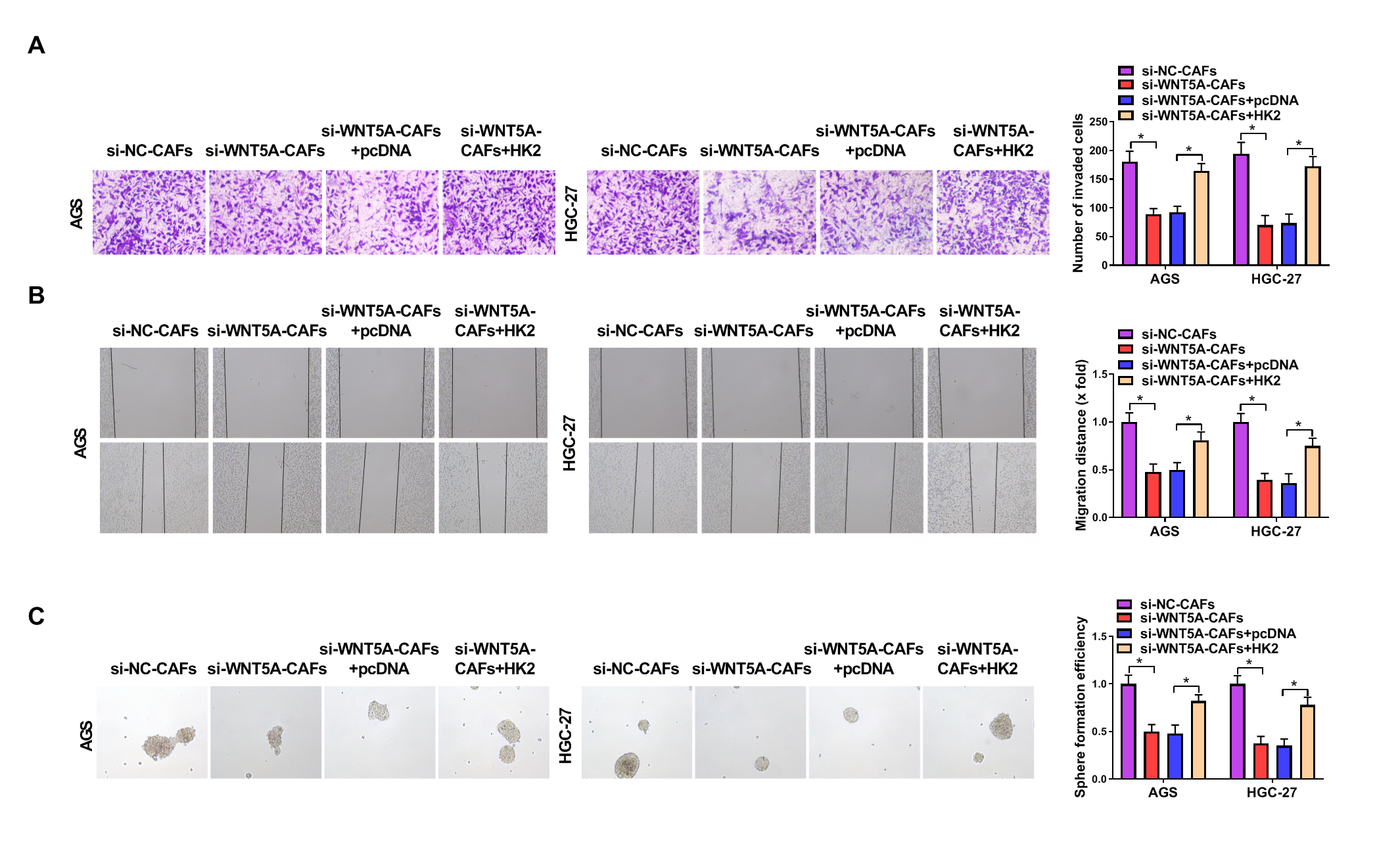

Supplement: Supplementary file 5 — Supplementary Material 5 [file 12957_2024_3482_MOESM5_ESM.png]

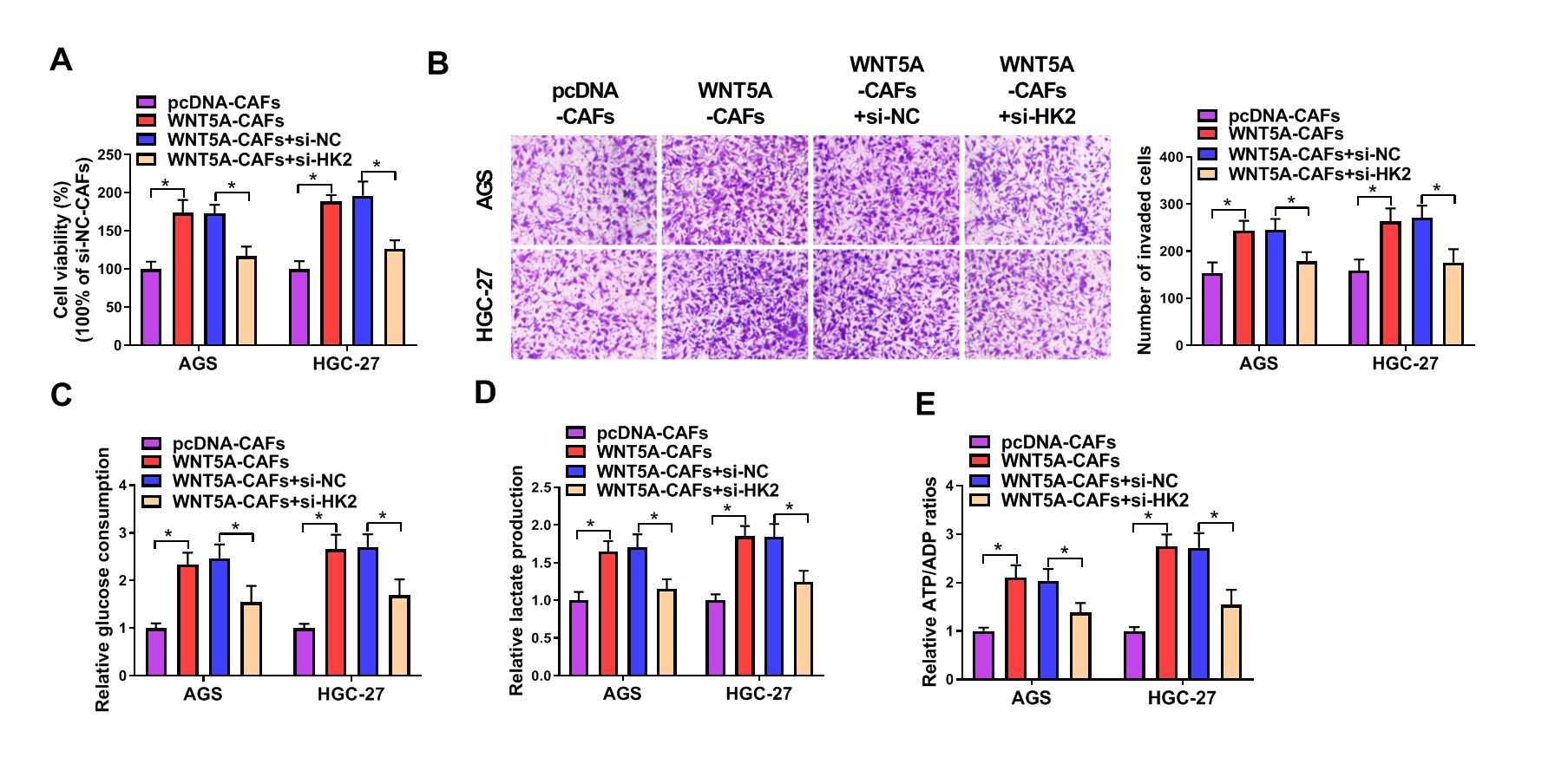

Supplement: Supplementary file 6 — Supplementary Material 6 [file 12957_2024_3482_MOESM6_ESM.png]
